# Association of Derived Neutrophil-To-Lymphocyte Ratio With Prognosis of Coronary Heart Disease After PCI

**DOI:** 10.3389/fcvm.2021.705862

**Published:** 2021-09-17

**Authors:** Gang-Qiong Liu, Wen-Jing Zhang, Jia-Hong Shangguan, Xiao-Dan Zhu, Wei Wang, Qian-Qian Guo, Jian-Chao Zhang, Kai Wang, Zhi-Yu Liu, Feng-Hua Song, Lei Fan, Ying-Ying Zheng, Jin-Ying Zhang

**Affiliations:** ^1^Department of Cardiology, Key Laboratory of Cardiac Injury and Repair of Henan Province, First Affiliated Hospital of Zhengzhou University, Zhengzhou, China; ^2^Administration Department of Henan Medical Association, Zhengzhou, China

**Keywords:** derived neutrophil-to-lymphocyte ratio, percutaneous coronary intervention, mortality, neutrophil-to-lymphocyte ratio, coronary heart disease

## Abstract

**Aims:** The present study aimed to investigate the prognostic role of derived neutrophil-to-lymphocyte ratio (dNLR) in patients with coronary heart disease (CHD) after PCI.

**Methods:** A total of 3,561 post-PCI patients with CHD were retrospectively enrolled in the CORFCHD-ZZ study from January 2013 to December 2017. The patients (3,462) were divided into three groups according to dNLR tertiles: the first tertile (dNLR < 1.36; *n* = 1,139), second tertile (1.36 ≥ dNLR < 1.96; *n* = 1,166), and third tertile(dNLR ≥ 1.96; *n* = 1,157). The mean follow-up time was 37.59 ± 22.24 months. The primary endpoint was defined as mortality (including all-cause death and cardiac death), and the secondary endpoint was major adverse cardiovascular events (MACEs) and major adverse cardiovascular and cerebrovascular events (MACCEs).

**Results:** There were 2,644 patients with acute coronary syndrome (ACS) and 838 patients with chronic coronary syndrome (CCS) in the present study. In the total population, the all-cause mortality (ACM) and cardiac mortality (CM) incidence was significantly higher in the third tertile than in the first tertile [hazard risk (HR) = 1.8 (95% CI: 1.2–2.8), *p* = 0.006 and HR = 2.1 (95% CI: 1.23–3.8), *p* = 0.009, respectively]. Multivariate Cox regression analyses suggested that compared with the patients in the first tertile than those in the third tertile, the risk of ACM was increased 1.763 times (HR = 1.763, 95% CI: 1.133–2.743, *p* = 0.012), and the risk of CM was increased 1.763 times (HR = 1.961, 95% CI: 1.083–3.550, *p* = 0.026) in the higher dNLR group during the long-term follow-up. In both ACS patients and CCS patients, there were significant differences among the three groups in the incidence of ACM in univariate analysis. We also found that the incidence of CM was significantly different among the three groups in CCS patients in both univariate analysis (HR = 3.541, 95% CI: 1.154–10.863, *p* = 0.027) and multivariate analysis (HR = 3.136, 95% CI: 1.015–9.690, *p* = 0.047).

**Conclusion:** The present study suggested that dNLR is an independent and novel predictor of mortality in CHD patients who underwent PCI.

## Introduction

Previous studies suggested that systemic inflammation is a predictive marker of clinical outcome of coronary heart disease (CHD) (1–5). Some biomarkers such as C-reactive protein (6, 7) and blood routine test parameters including leukocyte (8, 9) and neutrophil (10–12), which represent systemic inflammation, have been reported to be associated with adverse outcomes of CHD patients. Recently, a novel biomarker, derived neutrophil-to-lymphocyte ratio (dNLR), which was defined as neutrophil count/(leukocyte count—neutrophil count) has been demonstrated to be a predictor of chronic inflammatory diseases and cancers (13–18). Although CHD is a chronic inflammatory disease, a few studies have investigated the relation between dNLR and the long-term outcomes in CHD patients who underwent PCI. In the present study, we aimed to investigate the association of dNLR with the long-term outcomes in Chinese patients with angiographic CHD who underwent PCI.

## Methods

### Study Design and Population

In the present study, we enrolled 3,561 CHD patients who underwent PCI from January 2013 to December 2017 in the First Affiliated Hospital of Zhengzhou University. All the participants were CHD patients who underwent coronary angiograph and at least one stent being implanted. Both ACS (*n* = 2,644) and CCS (*n* = 838) were involved in this study. The diagnostic criteria of ACS were described previously. ACS includes unstable angina, ST segment elevation myocardial infarction, and non-ST segment elevation myocardial infarction. All the patients were administered with standard-dose double antiplatelet, statins, and β receptor blockers before PCI if there are no contraindications.

The patients with serious heart failure, hematological diseases, acute infections, and malignant tumor were excluded from the study. The Declaration of Helsinki was complied in the present study, and the protocol was approved by the ethics committee of the First Affiliated Hospital of Zhengzhou University. The need to obtain informed consent from eligible patients was waived by the ethics committee for the retrospective design of the study.

**Figure 1:**
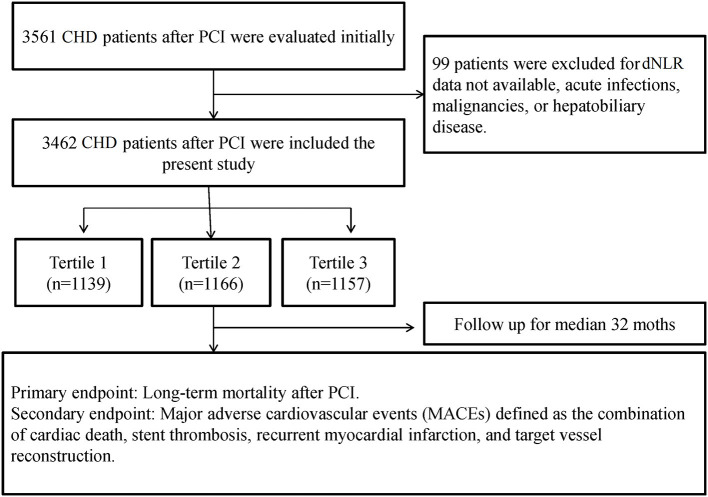
The follow chart of participant inclusion.

Ninety-nine patients were excluded due to hematologic parameters (data not available) or acute infections. Finally, there were 3,462 patients who were enrolled to the present study. A flowchart of inclusion and exclusion of patients is shown in Figure 1.

### Definition of Derived Neutrophil-To-Lymphocyte Ratio

The basal blood collection was performed before PCI. Routine blood testing performed in accordance with standard methods was as described previously. Briefly, 2 ml of venous blood samples were collected in standardized dipotassium EDTA tubes. The blood routine test was measured using an automated blood counter within 2 h of collection to minimize variations due to sample aging. We calculated the dNLR according to the formula as follows: neutrophil count/(leukocyte count—neutrophil count) as described previously (13–15).

### Endpoints

In the present study, we defined a primary endpoint and several secondary endpoints. The primary endpoint was the long-term mortality, including all-cause death and cardiac death. The secondary endpoints include major adverse cardiac events (MACE) and the major adverse cardiac and cerebrovascular events (MACCEs) as described previously (19–21).

### Follow-Up

We carried out the follow-up at least 18 months for each patient. The longest follow-up time is 80 months. All the patients were followed either by office visits or by telephone contacts as necessary.

### Statistical Analyses

The SPSS 22.0 for windows statistical software (SPSS Inc., Chicago, IL, USA) was used to analyze the data. The continuous data were presented as mean ± standard deviation (mean ± SD). The *t*-test was utilized to analyze the differences between normally distributed numeric variables, while the Mann–Whitney U-test was used to analyze the difference between non-normally distributed variables. The categorical data were presented as frequencies and percentages. Chi-square (χ^2^) test was employed for the comparison of categorical variables. The patients were divided into three groups according to the dNLR tertiles: the first tertile (dNLR < 1.36; *n* = 1,139), second tertile (1.36 ≥ dNLR < 1.96; *n* = 1,166), and third tertile dNLR ≥ 1.96; *n* = 1,157). We utilized the Kaplan–Meier analysis to analyze the cumulative incidence of long-term outcomes among three groups. The log-rank test was used to compare the difference between each group. To adjust the confounders, we used the multivariable Cox regression analysis to evaluate the predictive value of dNLR and to calculate the hazard ratios (HRs) and its 95% confidence intervals (CIs). Two-side *p* < 0.05 was considered significant.

## Result

### Baseline Characteristics of Three Groups

The study included 3,462 patients who were divided into three groups according to the dNLR tertiles: the first tertile (dNLR < 1.36; *n* = 1,139), second tertile (1.36 ≥ dNLR < 1.96; *n* = 1,166), and third tertile (dNLR ≥ 1.96; n = 1,157). The baseline data are shown in Table 1. In the total patients, there were significant differences among these three tertiles in dNLR, sex, white blood cell (WBC), creatinine (Cr), triglyceride (TG), total cholesterol (TC), high-density lipoprotein cholesterol (HDL-C), and smoking (all *p* < 0.05). We did not find a significant difference among these three groups in age, hypertension, diabetes mellitus, and alcohol drinking. We also showed the baseline data characteristics of ACS and CCS patients in Table 1.

**Table 1:** Characteristics of participants of the three groups.

**Variables**	**Total (*n* = 3,462)**	**Tertile 1 (<1.36) (*n* = 1,139)**	**Tertile 2 (1.36–1.96) (*n* = 1,166)**	**Tertile 3 (≥1.96) (*n* = 1,157)**	***p*-Value**
**Total population**					
dNLR	1.973 ± 1.727	1.078 ± 0.221	1.640 ± 0.168	3.217 ± 2.340	<0.001
Sex, female, *n* (%)	1,100 (30.9)	427 (35.7)	347 (29.8)	300 (25.9)	<0.001
Age, year	63.238 ± 10.615	63.213 ± 10.397	63.031 ± 10.491	63.561 ± 10.829	0.471
WBC (× 10^9^/L)	7.009 ± 2.239	6.054 ± 1.539	6.633 ± 1.569	8.329 ± 2.722	<0.001
Cr (μmol/L)	72.874 ± 35.63	69.500 ± 19.54	72.520 ± 25.192	76.505 ± 53.016	<0.001
TG (mmol/L)	1.662 ± 1.118	1.720 ± 1.244	1.670 ± 1.109	1.594 ± 0.979	0.031
TC (mmol/L)	3.903 ± 1.024	3.970 ± 1.031	3.8 50 ± 1.008	3.9 02 ± 1.023	0.020
HDL-C (mmol/L)	1.039 ± 0.293	1.060 ± 0.285	1.028 ± 0.323	1.031 ± 0.267	0.020
LDL-C (mmol/L)	2.397 ± 0.845	2.414 ± 0.845	2.357 ± 0.811	2.416 ± 0.874	0.176
Hypertension (*n*, %)	1,973 (55.4)	619 (54.3)	660 (56.6)	637 (55.1)	0.536
Diabetes (*n*, %)	838 (23.5)	254 (22.3)	277 (23.8)	292 (25.2)	0.255
Smoking (*n*, %)	1,083 (30.4)	305 (26.8)	370 (31.7)	383 (33.1)	0.003
Alcohol drinking (*n*, %)	558 (16.1)	159 (14.0)	203 (17.4)	196 (16.9)	0.051
AF (*n*, %)	46 (1.3)	14 (1.2)	10 (0.9)	22 (1.9)	0.084
Number of lesions (*n*, %)					0.295
1-VD	494 (13.9)	167 (14.5)	169 (14.4)	138 (11.9)	
2-VD	841 (23.6)	273 (23.8)	276 (23.6)	273 (23.5)	
3-VD	2,226 (62.5)	709 (61.7)	725 (62.0)	752 (64.7)	
LM	415 (11.9)	142 (12.4)	142 (12.1)	131 (11.3)	0.691
Medications (*n*, %)					
Aspirin	3,462 (100.00)	1,139 (100.00)	1,166 (100.00)	1,157 (100.00)	0.999
Clopidogrel or ticagrelor	3,462 (100.00)	1,139 (100.00)	1,166 (100.00)	1,157 (100.00)	0.999
β-blockors	2,474 (71.05)	792 (68.92)	843 (72.05)	839 (72.14)	0.053
Statins	3,468 (99.6)	1,145 (99.7)	1,162 (99.3)	1,161 (99.8)	0.139
**ACS patients**					
dNLR	2.00 ±1.87	1.04 ± 0.62	1.64 ± 0.18	3.32 ± 2.70	<0.001
Sex, female, *n* (%)	824 (31.2)	330 (38.3)	266 (29.5)	228 (25.9)	<0.001
Age, year	63.4 ± 10.4	63.3 ± 10.3	63.4 ± 10.3	63.6 ± 10.6	0.800
WBC (× 10^9^/L)	7.03 ± 2.29	6.02 ± 1.48	6.63 ± 10.34	8.42 ± 2.83	<0.001
Cr (μmol/L)	72.3 ± 25.5	70.2 ± 21.1	71.9 ± 18.2	74.7 ± 34.2	0.001
TG (mmol/L)	1.65 ± 1.09	1.72 ± 1.19	1.63 ± 1.08	1.59 ± 0.99	0.037
TC (mmol/L)	3.90 ± 1.03	3.96 ± 1.02	3.86 ± 1.06	3.90 ± 1.03	0.126
HDL-C (mmol/L)	1.04 ± 0.29	1.05 ± 0.27	1.03 ± 0.34	1.03 ± 0.26	0.335
LDL-C (mmol/L)	2.40 ± 0.85	2.41 ± 0.84	2.36 ± 0.83	2.43 ± 0.88	0.191
Hypertension (*n*, %)	1,474 (55.7)	480 (55.7)	509 (56.5)	485 (55.1)	0.828
Diabetes(*n*, %)	633 (23.9)	186 (21.6)	216 (24.0)	231 (26.2)	0.076
Smoking (*n*, %)	799 (30.2)	232 (26.9)	287 (31.9)	280 (31.8)	0.036
Alcohol drinking (*n*, %)	424 (16.0)	117 (13.6)	158 (17.5)	149 (16.9)	0.059
AF (*n*, %)	31 (1.2)	9 (1.0)	8 (0.8)	14 (1.5)	0.110
Number of lesions (*n*, %)					0.676
1-VD	352 (13.3)	118 (13.7)	126 (14.0)	108 (12.3)	
2-VD	616 (23.3)	207 (24.0)	211 (23.4)	198 (22.5)	
3-VD	1,676 (63.4)	537 (62.3)	564 (62.6)	575 (65.3)	
LM	322 (12.2)	114 (13.2)	107 (11.9)	101 (11.5)	0.502
Medications (*n*, %)					
Aspirin	2,644 (100.00)	862 (100.00)	901 (100.00)	881 (100.00)	-
Clopidogrel or ticagrelor	2,644 (100.00)	862 (100.00)	901 (100.00)	881 (100.00)	-
β-blockors	1,911 (92.9)	599 (91.5)	659 (92.4)	653 (94.9)	0.037
Statins	2,633 (99.6)	858 (99.5)	895 (99.3)	880 (99.9)	0.187
**CCS patients**					
dNLR	1.88 ± 1.18	1.03 ± 0.64	1.65 ± 0.17	2.96 ± 1.32	<0.001
Sex, female, *n* (%)	259 (30.9)	101 (35.2)	83 (30.9)	75 (26.6)	0.085
Age, year	62.7 ± 11.1	62.8 ± 1.7	61.9 ± 10.9	63.4 ± 11.6	0.292
WBC (× 10^9^/L)	6.95 ± 2.07	6.13 ± 1.69	6.66 ± 1.60	8.06 ± 2.32	<0.001
Cr (μmol/L)	74.7 ± 57.4	67.9 ± 15.4	74.4 ± 40.8	81.8 ± 78.5	0.017
TG (mmol/L)	1.72 ± 1.20	1.72 ± 1.40	1.81 ± 1.18	1.63 ± 0.95	0.219
TC (mmol/L)	3.90 ± 1.02	3.99 ± 1.06	3.82 ± 0.96	3.87 ± 1.02	0.159
HDL-C (mmol/L)	1.05 ± 0.29	1.09 ± 0.30	1.02 ± 0.27	1.04 ± 0.29	0.019
LDL-C (mmol/L)	2.39 ± 0.81	2.41 ± 0.84	2.36 ± 0.75	2.39 ± 0.85	0.748
Hypertension (*n*, %)	455 (54.3)	145 (50.5)	155 (57.6)	155 (55.0)	0.235
Diabetes (*n*, %)	192 (22.9)	66 (23.0)	62 (23.0)	64 (22.7)	0.994
Smoking (*n*, %)	263 (31.4)	74 (25.8)	84 (31.2)	105 (37.2)	0.013
Alcohol drinking (*n*, %)	137 (16.3)	43 (15.0)	45 (16.7)	49 (17.4)	0.727
AF(*n*, %)	15 (1.8)	5 (1.7)	6 (2.2)	4 (1.4)	0.770
Number of lesions (*n*, %)					0.228
1-VD	122 (14.6)	49 (17.1)	43 (16.0)	30 (10.6)	
2-VD	206 (24.6)	66 (23.0)	65 (24.2)	75 (26.6)	
3-VD	510 (60.9)	172 (59.9)	161 (59.9)	177 (62.8)	
LM	93 (11.1)	28 (9.8)	35 (13.0)	30 (10.6)	0.453
Medications (*n*, %)					
Aspirin	838 (100.0)	287 (100.0)	269 (100.0)	282 (100.0)	-
Clopidogrel or ticagrelor	838 (100.0)	287 (100.0)	269 (100.0)	282 (100.0)	-
β-blockors	563 (94.6)	193 (94.1)	184 (92.9)	186 (96.9)	0.210
Statins	835 (99.6)	287 (100.0)	267 (99.3)	281 (99.6)	0.341

*WBC, white blood cell; Cr, creatinine; TG, triglyceride; TC, total cholesterol; LDL-C, low-density lipoprotein cholesterol; HDL-C, high-density lipoprotein cholesterol; ACS, acute coronary syndrome*.

### Comparison of Clinical Outcomes Among Groups

Table 2 shows the incidence of ACM in the three groups. In the total population, there were 33 (2.9%) ACMs in the first tertile dNLR group and 57 (4.9%) ACMs in the third tertile group. The difference was significant (*p* = 0.026). We did not find a significant difference between the three groups in the incidence of CM, MACEs, and MACCEs. In ACS patients, the incidence of ACM was higher in the third tertile group than that in the first tertile group (4.5 vs. 3.0%, *p* = 0.048). In the CCS patients, both ACM (6.4 vs. 2.4%, *p* = 0.017) and CM (4.6 vs. 1.4%, *p* = 0.036) had significant frequency in the third tertile group than that in the first tertile group.

**Table 2:** Outcome comparison between groups.

**Outcomes**	**Tertile 1**	**Tertile 2**	**Tertile 3**	**p-Values**
	**(<1.36)**	**(1.36–1.96)**	**(≥1.96)**	
**Total patients**				
ACM (*n*, %)	33 (2.9)	39 (3.3)	57 (4.9)	0.026
CM (*n*, %)	18 (1.6)	28 (2.4)	36 (3.1)	0.054
MACE (*n*, %)	132 (11.6)	123 (10.5)	135 (11.7)	0.636
MACCE (*n*, %)	164 (14.4)	168 (14.4)	177 (15.3)	0.782
**ACS patients**				
ACM (*n*, %)	26 (3.0)	30 (3.3)	40 (4.5)	0.048
CM (*n*, %)	14 (1.6)	23 (2.6)	23 (2.6)	0.300
MACE (*n*, %)	102 (11.8)	92 (10.2)	99 (11.2)	0.546
MACCE (*n*, %)	124 (14.4)	125 (13.9)	135 (15.3)	0.679
**CCS patients**				
ACM (*n*, %)	7 (2.4)	9 (3.3)	18 (6.4)	0.017
CM (*n*, %)	4 (1.4)	5 (1.9)	13 (4.6)	0.036
MACE (*n*, %)	34 (11.8)	31 (11.5)	37 (13.1)	0.831
MACCE (*n*, %)	45 (15.7)	43 (16.0)	43 (15.2)	0.972

*ACM, all-cause mortality; CM, cardiac mortality; MACE, major adverse cardiovascular events; MACCE, major adverse cardiovascular and cerebrovascular events; ACS, acute coronary syndrome; CCS, chronic coronary syndrome*.

**Figure 2:**
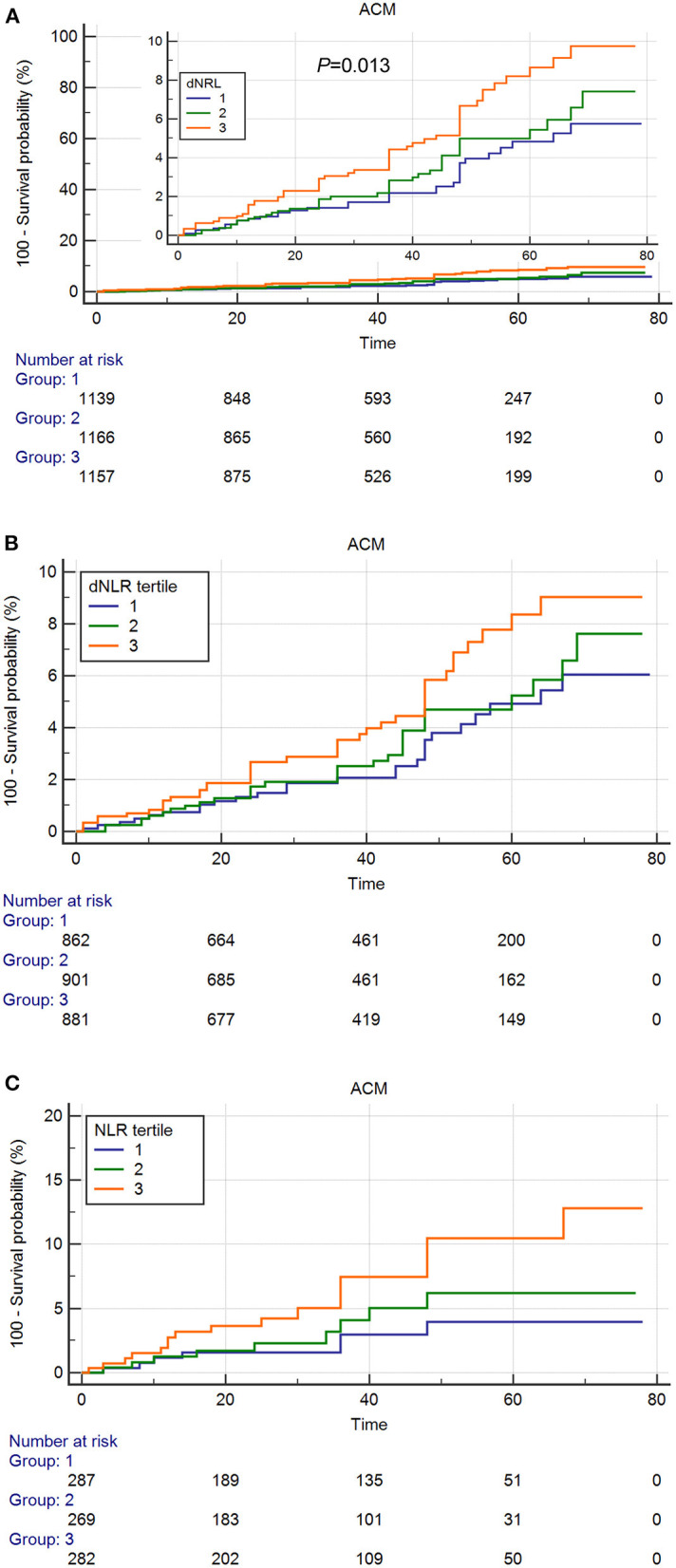
Cumulative Kaplan–Meier estimates of the time to the first adjudicated occurrence of all-cause death. **(A)** Total patients. **(B)** Acute coronary syndrome (ACS) patients. **(C)** Chronic coronary syndrome (CCS) patients.

**Figure 3:**
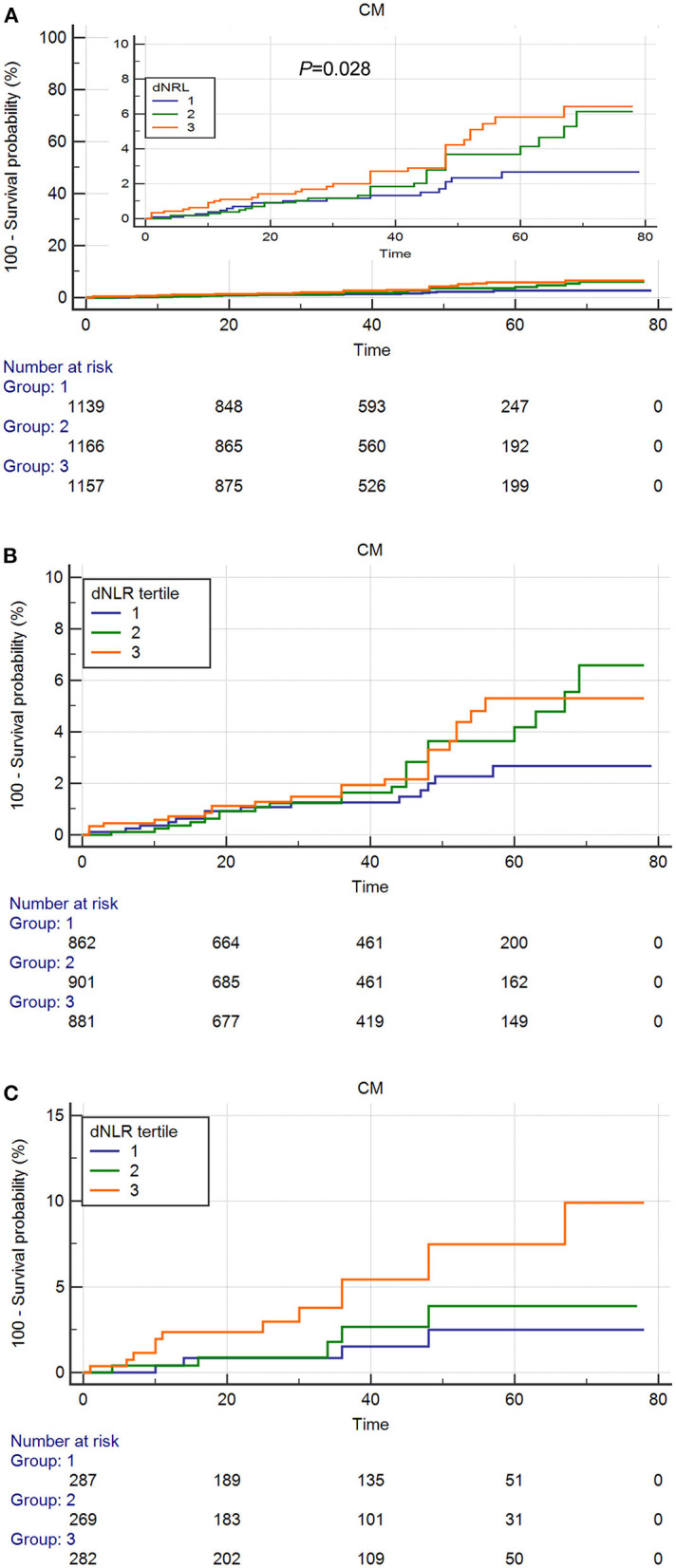
Cumulative Kaplan–Meier estimates of the time to the first adjudicated occurrence of cardiac death. **(A)** Total Patients. **(B)** ACS patients. **(C)** CCS patients.

As shown in the Figures 2, 3, the Kaplan–Meier survival analysis showed that patients in the third tertile have an increased risk of ACM and CM, compared with patients in the first tertile. This trend was also found in both ACS patients (Figures 2B,C) and CCS patients (Figures 3B,C).

As shown in Table 3, in the total patients, the ACM and CM incidence was significantly lower in the first tertile than that in the third tertile [HR = 1.8 (95% CI: 1.2–2.8), *p* = 0.006 and HR = 2.1 (95% CI: 1.23–3.8), *p* = 0.009, respectively]. Multivariate Cox regression analyses suggested that compared with the patients in the first tertile, the risk of ACM was increased 1.763 times (HR = 1.763, 95% CI: 1.133–2.743, *p* = 0.012), and the risk of CM was increased 1.961 times (HR = 1.961, 95% CI: 1.083–3.550, *p* = 0.026) in the third tertile dNLR group during the long-term follow-up. All individual dNLR values for survival or not survival are shown in Supplementary Table 1. In both ACS patients and CCS patients, there were significant differences among the three groups in the incidence of ACM in the univariate Cox regression analysis. We also found that the incidence of CM was significantly different among the three groups in the CCS patients in both univariate Cox regression analysis (HR = 3.541, 95% CI: 1.154–10.863, *p* = 0.027) and multivariate Cox regression analysis (HR = 3.136, 95% CI: 1.015–9.690, *p* = 0.047).

**Table 3:** Univariate and multivariable Cox regression analysis for outcomes.

**Outcomes**	**Non-adjusted**		**Adjusted**	
	**HR (95% CI)**	***p*-Value**	**HR (95% CI)**	***p-*Value**
**Total patients**				
ACM				
dNLR tertile				
1	1		1	
2	1.2 (0.8–2.0)	0.368	1.283 (0.803–2.049)	0.298
3	1.8 (1.2–2.8)	0.006	1.763 (1.133–2.743)	0.012
CM				
dNLR tertile				
1	1		1	
2	1.6 (0.9–3.0)	0.104	1.729 (0.945–3.164)	0.076
3	2.1 (1.2–3.8)	0.009	1.961 (1.083–3.550)	0.026
MACE				
dNLR tertile				
1	1		1	
2	1.0 (0.8–1.3)	0.956	1.0 (0.8–1.3)	0.777
3	1.1 (0.9–1.4)	0.483	1.0 (0.7–1.3)	0.864
MACCE				
dNLR tertile				
1	1		1	
2	1.1 (0.9–1.4)	0.36	1.1 (0.9–1.4)	0.457
3	1.2 (0.9–1.4)	0.165	1.0 (0.8–1.3)	0.929
**ACS patients**				
ACM				
dNLR tertile				
1	1		1	
2	1.180 (0.697–1.995)	0.538	1.144 (0.676–1.938)	0.616
3	1.643 (1.002–3.693)	0.049	1.472 (0.894–2.423)	0.129
CM				
dNLR tertile				
1	1		1	
2	1.684 (0.866–3.274)	0.124	1.559 (0.801–3.035)	0.191
3	1.755 (0.903–3.411)	0.097	1.462 (0.749–2.854)	0.266
MACE				
dNLR tertile				
1	1		1	
2	0.946 (0.713–1.255)	0.700	0.939 (0.707–1.246)	0.661
3	1.051 (0.797–1.387)	0.723	1.033 (0.782–1.366)	0.818
MACCE				
dNLR tertile				
1	1		1	
2	1.070 (0.834–1.373)	0.593	1.069 (0.833–1.373)	0.457
3	1.196 (0.937–1.528)	0.150	1.184 (0.926–1.515)	0.178
**CCS patients**				
ACM				
dNLR tertile				
1	1		1	
2	1.500 (0.558–4.030)	0.421	1.395 (0.518–3.756)	0.511
3	2.790 (1.165–6.683)	0.006	2.385 (1.010–5.748)	0.044
CM				
dNLR tertile				
1	1		1	
2	1.479 (0.397, 5.511)	0.560	1.403 (0.376–5.241)	0.615
3	3.541 (1.154, 10.863)	0.027	3.136 (1.015–9.690)	0.047
MACE				
dNLR tertile				
1	1		1	
2	1.081 (0.664–1.760)	0.754	1.078 (0.662–1.757)	0.762
3	1.162 (0.729–1.851)	0.529	1.161 (0.727–1.855)	0.532
MACCE				
dNLR tertile				
1	1		1	
2	1.146 (0.754–1.742)	0.523	1.142 (0.751–1.737)	0.535
3	1.015 (0.668–1.542)	0.944	1.007 (0.661–1.534)	0.973

*ACM, all-cause mortality; CM, cardiac mortality; MACE, major adverse cardiovascular events; MACCE, major adverse cardiovascular and cerebrovascular events; ACS, acute coronary syndrome; CCS, chronic coronary syndrome; dNLR, derived neutrophil-to-lymphocyte ratio*.

## Discussion

This study suggested that increased dNLR was independently associated with the prognosis of CHD patients who underwent PCI. This is the first study to investigate the relation between dNLR and prognosis in CHD patients.

The circulating cytokines and chemokines were released from elevated neutrophil and during inflammatory responses during which the counts of lymphocyte were declined. The proinflammatory markers including oxidized LDL, proinflammatory cytokines, adhesion molecules, serum amyloid A (SAA), and C-reactive protein (CRP) play an important role in the progress of atherosclerosis (3–5). Therefore, a large number of studies have focused on the relation between inflammatory markers and CHD (2–4). In our study, we used the dNLR, which was calculated as neutrophil count/(leukocyte count—neutrophil count) to predict the mortality of CHD patients who underwent PCI, and found that increased dNLR was associated with high mortality. Neutrophilia reflects the inflammation and the lymphocyte reflects the stress response in human body. Therefore, the dNLR represents a balance between these two paths (22, 23). In our study, we enrolled 3,462 CHD patients who underwent PCI and divided them into three groups according to the dNLR tertiles: the first tertile (dNLR < 1.36; *n* = 1,139), the second tertile (1.36 ≥ dNLR < 1.96; *n* = 1,166), and the third tertile (dNLR ≥ 1.96; *n* = 1,157). We found that the patients in the third tertile have higher mortality. After multivariable Cox regression analysis to adjust the confounders, the dNLR remains an independent predictor of mortality of CHD patients who underwent PCI. In addition, we also found that kidney function is the worst in the third tertile group, which may be an important impact factor of outcomes. A previous study (24) showed that NLR was significantly increased in patients with renal failure, which was in line with our results. Importantly, when we adjusted the Cr variable, the dNLR remains a significant predictive power of mortality.

Recently, Agarwal et al. (25) reviewed the prognostic value of NLR across all stages of CHD. In the review of Agarwal, three studies (26–28) reported that higher NLR was associated with increased risk of all-cause death or cardiac death. In addition, two studies (29, 30) from Boag et al. and Spray et al. with up to 5,000 patients showed clearly the impact of lymphopenia on mortality in STEMI patients. Theoretically, for STEMI patients, with the rise in NLR levels, serum troponin will also be increased correspondingly. Severe myocardial injury and ischemia will stimulate the hypothalamus to promote the release of adrenocortic hormones and to increase cortisol level. Cortisol can help to regulate the peripheral leukocyte count. Once the cortisol level increases *in vivo*, the number of neutral granulocytes will be improved, and the number of lymphocytes will be reduced accordingly, thereby increasing the level of NLR. In addition, lymphopenia is related to age. Also, approximately 70% of lymphocytes in the peripheral blood are produced in the thymus. Therefore, thymus functional degradation may affect the production of lymphocytes. In our study, we utilized multivariable Cox regression analysis to adjust age and other confounders, and dNLR remains a significant predictive value of outcomes.

Several mechanistic pathways may connect NLR to adverse prognosis in CHD patients. First, previous studies (25, 31)suggested that increased neutrophils can result in the production of vascular endothelial growth factor (VEGF), IL-1, IL-6, and IL-17, which all were reported to be associated with adverse outcomes of CHD. Second, lymphocytes have been suggested to play a role in the modulation of the inflammatory response throughout the atherosclerotic process (32). Lymphopenia may be aggravated by redistribution of T cells from the circulation to lymphoid tissues (33); it may induce compensatory proliferation of antigen-experienced T cells, which could increase the risk of cardiovascular disease (34). Third, lymphocytes have a critical role in regulating the inflammatory response to tissue damage, through production of both pro- and anti-inflammatory cytokines.

There were several strengths in our study. First, the long-term follow-up is the main strength of our study. Second, the larger sample size is another strength of our study. Finally, we performed the statistical analysis that used multivariable analysis, which improved the credibility of the study. In addition, there were also some limitations, which are as follows: First, we only collected the baseline data of dNLR; therefore, the influence of dynamic changes of dNLR on the mortality was unclear. Second, there were several patients who were excluded from this study for data not available. Third, our study is a single-center retrospective cohort design; therefore, a multicenter cohort design is needed in the future. Fourth, all the patients were not continuously included in this study, which will be of the selection bias. Finally, we did not collect the revascularization characteristics in this study. Therefore, we cannot analyze the impact of revascularization on outcomes in the present study.

## Conclusion

In conclusion, our results suggest that baseline dNLR is a novel independent predictor of mortality in CHD patients who underwent PCI. This founding will help the interventional physician to make clinical decisions on daily practice.

## Data Availability Statement

The raw data supporting the conclusions of this article will be made available by the authors, without undue reservation.

## Ethics Statement

The studies involving human participants were reviewed and approved by The ethics committee of the First Affiliated Hospital of Zhengzhou University. The patients/participants provided their written informed consent to participate in this study.

## Author Contributions

G-QL and W-JZ made substantial contributions to study conception and design and to the drafting and critical revision of the manuscript for important intellectual content. J-HS, X-DZ, WW, Q-QG, J-CZ, and KW made substantial contributions to the study conception and design and critical revision of the manuscript for important intellectual content. Z-YL, F-HS, and LF made substantial contributions to study conception and design. Y-YZ and J-YZ made substantial contributions to study conception and design, drafting and critical revision of the manuscript for important intellectual content, including study supervision. All authors read and approved the final manuscript.

## Funding

This research was funded by the National Natural Science Foundation of China (U2004203, 81900263, and 81760043).

## Conflict of Interest

The authors declare that the research was conducted in the absence of any commercial or financial relationships that could be construed as a potential conflict of interest.

## Publisher's Note

All claims expressed in this article are solely those of the authors and do not necessarily represent those of their affiliated organizations, or those of the publisher, the editors and the reviewers. Any product that may be evaluated in this article, or claim that may be made by its manufacturer, is not guaranteed or endorsed by the publisher.

## Abbreviations

dNLR: derived neutrophil-to-lymphocyte ratio
PCI: percutaneous coronary intervention
CHD: coronary heart disease
MACE: major adverse cardiac events
MACCEs: major adverse cardiac and cerebrovascular events
HR: hazard ratios
CI: confidence interval.
