# Supplementary material for: Association of Derived Neutrophil-To-Lymphocyte Ratio With Prognosis of Coronary Heart Disease After PCI
Source: Front Cardiovasc Med. 2021 Sep 17;8:705862. doi: 10.3389/fcvm.2021.705862 (PMC8484317; doi:10.3389/fcvm.2021.705862)
Supplement: Supplementary file 1 [file Data_Sheet_1.docx]

| **Survival Table** | | | | | | | |
| --- | --- | --- | --- | --- | --- | --- | --- |
| dNLR | | Time | Status | Cumulative Proportion Surviving at the Time | | N of Cumulative Events | N of Remaining Cases |
|  |  |  |  | Estimate | Std. Error |  |  |
| .42 | 1 | 3.000 | 1.0 | .000 | .000 | 1 | 0 |
| .71 | 1 | 12.000 | 1.0 | .833 | .152 | 1 | 5 |
|  | 2 | 14.000 | .0 | . | . | 1 | 4 |
|  | 3 | 19.000 | .0 | . | . | 1 | 3 |
|  | 4 | 23.000 | .0 | . | . | 1 | 2 |
|  | 5 | 60.000 | .0 | . | . | 1 | 1 |
|  | 6 | 71.000 | .0 | . | . | 1 | 0 |
| .77 | 1 | 53.000 | 1.0 | .750 | .217 | 1 | 3 |
|  | 2 | 61.000 | .0 | . | . | 1 | 2 |
|  | 3 | 65.000 | .0 | . | . | 1 | 1 |
|  | 4 | 70.000 | .0 | . | . | 1 | 0 |
| .83 | 1 | 6.000 | 1.0 | .900 | .095 | 1 | 9 |
|  | 2 | 8.000 | .0 | . | . | 1 | 8 |
|  | 3 | 20.000 | .0 | . | . | 1 | 7 |
|  | 4 | 23.000 | .0 | . | . | 1 | 6 |
|  | 5 | 28.000 | .0 | . | . | 1 | 5 |
|  | 6 | 43.000 | .0 | . | . | 1 | 4 |
|  | 7 | 44.000 | .0 | . | . | 1 | 3 |
|  | 8 | 45.000 | .0 | . | . | 1 | 2 |
|  | 9 | 53.000 | .0 | . | . | 1 | 1 |
|  | 10 | 69.000 | .0 | . | . | 1 | 0 |
| .89 | 1 | 36.000 | 1.0 | .833 | .152 | 1 | 5 |
|  | 2 | 40.000 | .0 | . | . | 1 | 4 |
|  | 3 | 50.000 | .0 | . | . | 1 | 3 |
|  | 4 | 64.000 | .0 | . | . | 1 | 2 |
|  | 5 | 72.000 | .0 | . | . | 1 | 1 |
|  | 6 | 78.000 | .0 | . | . | 1 | 0 |
| .90 | 1 | 10.000 | 1.0 | .833 | .152 | 1 | 5 |
|  | 2 | 12.000 | .0 | . | . | 1 | 4 |
|  | 3 | 13.000 | .0 | . | . | 1 | 3 |
|  | 4 | 21.000 | .0 | . | . | 1 | 2 |
|  | 5 | 27.000 | .0 | . | . | 1 | 1 |
|  | 6 | 46.000 | .0 | . | . | 1 | 0 |
| .92 | 1 | 47.000 | 1.0 | .667 | .272 | 1 | 2 |
|  | 2 | 48.000 | .0 | . | . | 1 | 1 |
|  | 3 | 71.000 | .0 | . | . | 1 | 0 |
| .97 | 1 | 48.000 | 1.0 | .875 | .117 | 1 | 7 |
|  | 2 | 48.000 | .0 | . | . | 1 | 6 |
|  | 3 | 49.000 | .0 | . | . | 1 | 5 |
|  | 4 | 52.000 | .0 | . | . | 1 | 4 |
|  | 5 | 53.000 | .0 | . | . | 1 | 3 |
|  | 6 | 54.000 | .0 | . | . | 1 | 2 |
|  | 7 | 69.000 | .0 | . | . | 1 | 1 |
|  | 8 | 70.000 | .0 | . | . | 1 | 0 |
| 1.04 | 1 | 8.000 | 1.0 | .957 | .043 | 1 | 22 |
|  | 2 | 12.000 | .0 | . | . | 1 | 21 |
|  | 3 | 18.000 | .0 | . | . | 1 | 20 |
|  | 4 | 21.000 | .0 | . | . | 1 | 19 |
|  | 5 | 23.000 | .0 | . | . | 1 | 18 |
|  | 6 | 26.000 | .0 | . | . | 1 | 17 |
|  | 7 | 27.000 | .0 | . | . | 1 | 16 |
|  | 8 | 27.000 | .0 | . | . | 1 | 15 |
|  | 9 | 43.000 | .0 | . | . | 1 | 14 |
|  | 10 | 45.000 | .0 | . | . | 1 | 13 |
|  | 11 | 46.000 | .0 | . | . | 1 | 12 |
|  | 12 | 46.000 | .0 | . | . | 1 | 11 |
|  | 13 | 51.000 | .0 | . | . | 1 | 10 |
|  | 14 | 54.000 | .0 | . | . | 1 | 9 |
|  | 15 | 54.000 | .0 | . | . | 1 | 8 |
|  | 16 | 55.000 | .0 | . | . | 1 | 7 |
|  | 17 | 56.000 | .0 | . | . | 1 | 6 |
|  | 18 | 58.000 | .0 | . | . | 1 | 5 |
|  | 19 | 64.000 | .0 | . | . | 1 | 4 |
|  | 20 | 73.000 | .0 | . | . | 1 | 3 |
|  | 21 | 74.000 | .0 | . | . | 1 | 2 |
|  | 22 | 76.000 | .0 | . | . | 1 | 1 |
|  | 23 | 76.000 | .0 | . | . | 1 | 0 |
| 1.05 | 1 | 29.000 | 1.0 | .889 | .105 | 1 | 8 |
|  | 2 | 39.000 | .0 | . | . | 1 | 7 |
|  | 3 | 44.000 | .0 | . | . | 1 | 6 |
|  | 4 | 54.000 | .0 | . | . | 1 | 5 |
|  | 5 | 57.000 | .0 | . | . | 1 | 4 |
|  | 6 | 62.000 | .0 | . | . | 1 | 3 |
|  | 7 | 68.000 | .0 | . | . | 1 | 2 |
|  | 8 | 73.000 | .0 | . | . | 1 | 1 |
|  | 9 | 74.000 | .0 | . | . | 1 | 0 |
| 1.06 | 1 | 64.000 | 1.0 | .667 | .272 | 1 | 2 |
|  | 2 | 66.000 | .0 | . | . | 1 | 1 |
|  | 3 | 71.000 | .0 | . | . | 1 | 0 |
| 1.09 | 1 | 44.000 | 1.0 | .833 | .152 | 1 | 5 |
|  | 2 | 68.000 | .0 | . | . | 1 | 4 |
|  | 3 | 68.000 | .0 | . | . | 1 | 3 |
|  | 4 | 69.000 | .0 | . | . | 1 | 2 |
|  | 5 | 72.000 | .0 | . | . | 1 | 1 |
|  | 6 | 76.000 | .0 | . | . | 1 | 0 |
| 1.14 | 1 | 57.000 | 1.0 | .833 | .152 | 1 | 5 |
|  | 2 | 60.000 | .0 | . | . | 1 | 4 |
|  | 3 | 64.000 | .0 | . | . | 1 | 3 |
|  | 4 | 69.000 | .0 | . | . | 1 | 2 |
|  | 5 | 76.000 | .0 | . | . | 1 | 1 |
|  | 6 | 78.000 | .0 | . | . | 1 | 0 |
| 1.16 | 1 | 49.000 | 1.0 | .941 | .057 | 1 | 16 |
|  | 2 | 49.000 | .0 | . | . | 1 | 15 |
|  | 3 | 50.000 | .0 | . | . | 1 | 14 |
|  | 4 | 51.000 | .0 | . | . | 1 | 13 |
|  | 5 | 51.000 | .0 | . | . | 1 | 12 |
|  | 6 | 52.000 | .0 | . | . | 1 | 11 |
|  | 7 | 53.000 | .0 | . | . | 1 | 10 |
|  | 8 | 54.000 | .0 | . | . | 1 | 9 |
|  | 9 | 57.000 | .0 | . | . | 1 | 8 |
|  | 10 | 59.000 | .0 | . | . | 1 | 7 |
|  | 11 | 62.000 | .0 | . | . | 1 | 6 |
|  | 12 | 69.000 | .0 | . | . | 1 | 5 |
|  | 13 | 70.000 | .0 | . | . | 1 | 4 |
|  | 14 | 71.000 | .0 | . | . | 1 | 3 |
|  | 15 | 72.000 | .0 | . | . | 1 | 2 |
|  | 16 | 74.000 | .0 | . | . | 1 | 1 |
|  | 17 | 76.000 | .0 | . | . | 1 | 0 |
| 1.20 | 1 | 44.000 | 1.0 | .833 | .152 | 1 | 5 |
|  | 2 | 48.000 | .0 | . | . | 1 | 4 |
|  | 3 | 50.000 | .0 | . | . | 1 | 3 |
|  | 4 | 54.000 | .0 | . | . | 1 | 2 |
|  | 5 | 55.000 | .0 | . | . | 1 | 1 |
|  | 6 | 56.000 | .0 | . | . | 1 | 0 |
| 1.21 | 1 | 10.000 | 1.0 | .955 | .044 | 1 | 21 |
|  | 2 | 19.000 | .0 | . | . | 1 | 20 |
|  | 3 | 20.000 | .0 | . | . | 1 | 19 |
|  | 4 | 23.000 | .0 | . | . | 1 | 18 |
|  | 5 | 24.000 | .0 | . | . | 1 | 17 |
|  | 6 | 26.000 | .0 | . | . | 1 | 16 |
|  | 7 | 27.000 | .0 | . | . | 1 | 15 |
|  | 8 | 28.000 | .0 | . | . | 1 | 14 |
|  | 9 | 29.000 | .0 | . | . | 1 | 13 |
|  | 10 | 36.000 | .0 | . | . | 1 | 12 |
|  | 11 | 43.000 | .0 | . | . | 1 | 11 |
|  | 12 | 45.000 | .0 | . | . | 1 | 10 |
|  | 13 | 47.000 | .0 | . | . | 1 | 9 |
|  | 14 | 48.000 | .0 | . | . | 1 | 8 |
|  | 15 | 49.000 | .0 | . | . | 1 | 7 |
|  | 16 | 54.000 | .0 | . | . | 1 | 6 |
|  | 17 | 60.000 | .0 | . | . | 1 | 5 |
|  | 18 | 60.000 | .0 | . | . | 1 | 4 |
|  | 19 | 67.000 | .0 | . | . | 1 | 3 |
|  | 20 | 71.000 | .0 | . | . | 1 | 2 |
|  | 21 | 72.000 | .0 | . | . | 1 | 1 |
|  | 22 | 76.000 | .0 | . | . | 1 | 0 |
| 1.22 | 1 | 48.000 | 1.0 | .875 | .117 | 1 | 7 |
|  | 2 | 52.000 | .0 | . | . | 1 | 6 |
|  | 3 | 59.000 | .0 | . | . | 1 | 5 |
|  | 4 | 61.000 | .0 | . | . | 1 | 4 |
|  | 5 | 64.000 | .0 | . | . | 1 | 3 |
|  | 6 | 67.000 | .0 | . | . | 1 | 2 |
|  | 7 | 77.000 | .0 | . | . | 1 | 1 |
|  | 8 | 77.000 | .0 | . | . | 1 | 0 |
| 1.23 | 1 | 8.000 | 1.0 | .957 | .043 | 1 | 22 |
|  | 2 | 8.000 | .0 | . | . | 1 | 21 |
|  | 3 | 12.000 | .0 | . | . | 1 | 20 |
|  | 4 | 12.000 | .0 | . | . | 1 | 19 |
|  | 5 | 12.000 | .0 | . | . | 1 | 18 |
|  | 6 | 20.000 | .0 | . | . | 1 | 17 |
|  | 7 | 23.000 | .0 | . | . | 1 | 16 |
|  | 8 | 24.000 | .0 | . | . | 1 | 15 |
|  | 9 | 29.000 | .0 | . | . | 1 | 14 |
|  | 10 | 30.000 | .0 | . | . | 1 | 13 |
|  | 11 | 44.000 | .0 | . | . | 1 | 12 |
|  | 12 | 45.000 | .0 | . | . | 1 | 11 |
|  | 13 | 45.000 | .0 | . | . | 1 | 10 |
|  | 14 | 45.000 | .0 | . | . | 1 | 9 |
|  | 15 | 48.000 | .0 | . | . | 1 | 8 |
|  | 16 | 48.000 | .0 | . | . | 1 | 7 |
|  | 17 | 51.000 | .0 | . | . | 1 | 6 |
|  | 18 | 52.000 | .0 | . | . | 1 | 5 |
|  | 19 | 54.000 | .0 | . | . | 1 | 4 |
|  | 20 | 56.000 | .0 | . | . | 1 | 3 |
|  | 21 | 68.000 | .0 | . | . | 1 | 2 |
|  | 22 | 70.000 | .0 | . | . | 1 | 1 |
|  | 23 | 72.000 | .0 | . | . | 1 | 0 |
| 1.24 | 1 | 14.000 | 1.0 | .952 | .046 | 1 | 20 |
|  | 2 | 15.000 | .0 | . | . | 1 | 19 |
|  | 3 | 23.000 | .0 | . | . | 1 | 18 |
|  | 4 | 26.000 | .0 | . | . | 1 | 17 |
|  | 5 | 27.000 | .0 | . | . | 1 | 16 |
|  | 6 | 30.000 | .0 | . | . | 1 | 15 |
|  | 7 | 30.000 | .0 | . | . | 1 | 14 |
|  | 8 | 42.000 | .0 | . | . | 1 | 13 |
|  | 9 | 50.000 | .0 | . | . | 1 | 12 |
|  | 10 | 53.000 | .0 | . | . | 1 | 11 |
|  | 11 | 53.000 | .0 | . | . | 1 | 10 |
|  | 12 | 56.000 | .0 | . | . | 1 | 9 |
|  | 13 | 60.000 | .0 | . | . | 1 | 8 |
|  | 14 | 67.000 | .0 | . | . | 1 | 7 |
|  | 15 | 67.000 | .0 | . | . | 1 | 6 |
|  | 16 | 72.000 | .0 | . | . | 1 | 5 |
|  | 17 | 73.000 | .0 | . | . | 1 | 4 |
|  | 18 | 74.000 | .0 | . | . | 1 | 3 |
|  | 19 | 74.000 | .0 | . | . | 1 | 2 |
|  | 20 | 75.000 | .0 | . | . | 1 | 1 |
|  | 21 | 76.000 | .0 | . | . | 1 | 0 |
| 1.25 | 1 | 19.000 | 1.0 | .950 | .049 | 1 | 19 |
|  | 2 | 20.000 | .0 | . | . | 1 | 18 |
|  | 3 | 20.000 | .0 | . | . | 1 | 17 |
|  | 4 | 21.000 | .0 | . | . | 1 | 16 |
|  | 5 | 22.000 | .0 | . | . | 1 | 15 |
|  | 6 | 25.000 | .0 | . | . | 1 | 14 |
|  | 7 | 26.000 | .0 | . | . | 1 | 13 |
|  | 8 | 27.000 | .0 | . | . | 1 | 12 |
|  | 9 | 39.000 | .0 | . | . | 1 | 11 |
|  | 10 | 43.000 | .0 | . | . | 1 | 10 |
|  | 11 | 43.000 | .0 | . | . | 1 | 9 |
|  | 12 | 46.000 | .0 | . | . | 1 | 8 |
|  | 13 | 48.000 | .0 | . | . | 1 | 7 |
|  | 14 | 52.000 | .0 | . | . | 1 | 6 |
|  | 15 | 52.000 | .0 | . | . | 1 | 5 |
|  | 16 | 54.000 | .0 | . | . | 1 | 4 |
|  | 17 | 68.000 | .0 | . | . | 1 | 3 |
|  | 18 | 72.000 | .0 | . | . | 1 | 2 |
|  | 19 | 73.000 | .0 | . | . | 1 | 1 |
|  | 20 | 77.000 | .0 | . | . | 1 | 0 |
| 1.26 | 1 | 1.000 | 1.0 | .972 | .027 | 1 | 35 |
|  | 2 | 1.000 | .0 | . | . | 1 | 34 |
|  | 3 | 1.000 | .0 | . | . | 1 | 33 |
|  | 4 | 1.000 | .0 | . | . | 1 | 32 |
|  | 5 | 1.000 | .0 | . | . | 1 | 31 |
|  | 6 | 2.000 | .0 | . | . | 1 | 30 |
|  | 7 | 3.000 | 1.0 | .940 | .041 | 2 | 29 |
|  | 8 | 13.000 | .0 | . | . | 2 | 28 |
|  | 9 | 18.000 | .0 | . | . | 2 | 27 |
|  | 10 | 22.000 | .0 | . | . | 2 | 26 |
|  | 11 | 24.000 | .0 | . | . | 2 | 25 |
|  | 12 | 25.000 | .0 | . | . | 2 | 24 |
|  | 13 | 26.000 | .0 | . | . | 2 | 23 |
|  | 14 | 27.000 | .0 | . | . | 2 | 22 |
|  | 15 | 27.000 | .0 | . | . | 2 | 21 |
|  | 16 | 27.000 | .0 | . | . | 2 | 20 |
|  | 17 | 28.000 | .0 | . | . | 2 | 19 |
|  | 18 | 36.000 | .0 | . | . | 2 | 18 |
|  | 19 | 48.000 | .0 | . | . | 2 | 17 |
|  | 20 | 48.000 | .0 | . | . | 2 | 16 |
|  | 21 | 51.000 | .0 | . | . | 2 | 15 |
|  | 22 | 52.000 | .0 | . | . | 2 | 14 |
|  | 23 | 54.000 | .0 | . | . | 2 | 13 |
|  | 24 | 55.000 | .0 | . | . | 2 | 12 |
|  | 25 | 55.000 | .0 | . | . | 2 | 11 |
|  | 26 | 56.000 | .0 | . | . | 2 | 10 |
|  | 27 | 56.000 | .0 | . | . | 2 | 9 |
|  | 28 | 58.000 | .0 | . | . | 2 | 8 |
|  | 29 | 60.000 | .0 | . | . | 2 | 7 |
|  | 30 | 61.000 | .0 | . | . | 2 | 6 |
|  | 31 | 62.000 | .0 | . | . | 2 | 5 |
|  | 32 | 71.000 | .0 | . | . | 2 | 4 |
|  | 33 | 74.000 | .0 | . | . | 2 | 3 |
|  | 34 | 74.000 | .0 | . | . | 2 | 2 |
|  | 35 | 75.000 | .0 | . | . | 2 | 1 |
|  | 36 | 78.000 | .0 | . | . | 2 | 0 |
| 1.27 | 1 | 17.000 | 1.0 | .950 | .049 | 1 | 19 |
|  | 2 | 18.000 | .0 | . | . | 1 | 18 |
|  | 3 | 21.000 | .0 | . | . | 1 | 17 |
|  | 4 | 24.000 | .0 | . | . | 1 | 16 |
|  | 5 | 25.000 | .0 | . | . | 1 | 15 |
|  | 6 | 25.000 | .0 | . | . | 1 | 14 |
|  | 7 | 27.000 | .0 | . | . | 1 | 13 |
|  | 8 | 32.000 | .0 | . | . | 1 | 12 |
|  | 9 | 36.000 | .0 | . | . | 1 | 11 |
|  | 10 | 45.000 | .0 | . | . | 1 | 10 |
|  | 11 | 45.000 | .0 | . | . | 1 | 9 |
|  | 12 | 46.000 | .0 | . | . | 1 | 8 |
|  | 13 | 48.000 | .0 | . | . | 1 | 7 |
|  | 14 | 49.000 | .0 | . | . | 1 | 6 |
|  | 15 | 49.000 | .0 | . | . | 1 | 5 |
|  | 16 | 50.000 | .0 | . | . | 1 | 4 |
|  | 17 | 51.000 | .0 | . | . | 1 | 3 |
|  | 18 | 57.000 | .0 | . | . | 1 | 2 |
|  | 19 | 57.000 | .0 | . | . | 1 | 1 |
|  | 20 | 75.000 | .0 | . | . | 1 | 0 |
| 1.28 | 1 | 36.000 | 1.0 | .933 | .064 | 1 | 14 |
|  | 2 | 36.000 | .0 | . | . | 1 | 13 |
|  | 3 | 44.000 | .0 | . | . | 1 | 12 |
|  | 4 | 45.000 | .0 | . | . | 1 | 11 |
|  | 5 | 48.000 | .0 | . | . | 1 | 10 |
|  | 6 | 49.000 | .0 | . | . | 1 | 9 |
|  | 7 | 52.000 | .0 | . | . | 1 | 8 |
|  | 8 | 55.000 | .0 | . | . | 1 | 7 |
|  | 9 | 60.000 | .0 | . | . | 1 | 6 |
|  | 10 | 68.000 | .0 | . | . | 1 | 5 |
|  | 11 | 71.000 | .0 | . | . | 1 | 4 |
|  | 12 | 73.000 | .0 | . | . | 1 | 3 |
|  | 13 | 74.000 | .0 | . | . | 1 | 2 |
|  | 14 | 74.000 | .0 | . | . | 1 | 1 |
|  | 15 | 75.000 | .0 | . | . | 1 | 0 |
| 1.29 | 1 | 67.000 | 1.0 | .857 | .132 | 1 | 6 |
|  | 2 | 67.000 | .0 | . | . | 1 | 5 |
|  | 3 | 67.000 | .0 | . | . | 1 | 4 |
|  | 4 | 68.000 | .0 | . | . | 1 | 3 |
|  | 5 | 70.000 | .0 | . | . | 1 | 2 |
|  | 6 | 72.000 | .0 | . | . | 1 | 1 |
|  | 7 | 73.000 | .0 | . | . | 1 | 0 |
| 1.30 | 1 | 22.000 | 1.0 | .960 | .039 | 1 | 24 |
|  | 2 | 22.000 | .0 | . | . | 1 | 23 |
|  | 3 | 22.000 | .0 | . | . | 1 | 22 |
|  | 4 | 24.000 | .0 | . | . | 1 | 21 |
|  | 5 | 25.000 | .0 | . | . | 1 | 20 |
|  | 6 | 25.000 | .0 | . | . | 1 | 19 |
|  | 7 | 26.000 | .0 | . | . | 1 | 18 |
|  | 8 | 28.000 | .0 | . | . | 1 | 17 |
|  | 9 | 36.000 | 1.0 | .904 | .066 | 2 | 16 |
|  | 10 | 39.000 | .0 | . | . | 2 | 15 |
|  | 11 | 44.000 | .0 | . | . | 2 | 14 |
|  | 12 | 45.000 | .0 | . | . | 2 | 13 |
|  | 13 | 46.000 | .0 | . | . | 2 | 12 |
|  | 14 | 48.000 | .0 | . | . | 2 | 11 |
|  | 15 | 50.000 | .0 | . | . | 2 | 10 |
|  | 16 | 50.000 | .0 | . | . | 2 | 9 |
|  | 17 | 52.000 | .0 | . | . | 2 | 8 |
|  | 18 | 52.000 | .0 | . | . | 2 | 7 |
|  | 19 | 57.000 | .0 | . | . | 2 | 6 |
|  | 20 | 60.000 | .0 | . | . | 2 | 5 |
|  | 21 | 70.000 | .0 | . | . | 2 | 4 |
|  | 22 | 70.000 | .0 | . | . | 2 | 3 |
|  | 23 | 73.000 | .0 | . | . | 2 | 2 |
|  | 24 | 76.000 | .0 | . | . | 2 | 1 |
|  | 25 | 77.000 | .0 | . | . | 2 | 0 |
| 1.31 | 1 | 29.000 | 1.0 | .875 | .117 | 1 | 7 |
|  | 2 | 29.000 | .0 | . | . | 1 | 6 |
|  | 3 | 46.000 | .0 | . | . | 1 | 5 |
|  | 4 | 53.000 | .0 | . | . | 1 | 4 |
|  | 5 | 73.000 | .0 | . | . | 1 | 3 |
|  | 6 | 77.000 | .0 | . | . | 1 | 2 |
|  | 7 | 77.000 | .0 | . | . | 1 | 1 |
|  | 8 | 78.000 | .0 | . | . | 1 | 0 |
| 1.32 | 1 | 55.000 | 1.0 | .857 | .132 | 1 | 6 |
|  | 2 | 55.000 | .0 | . | . | 1 | 5 |
|  | 3 | 55.000 | .0 | . | . | 1 | 4 |
|  | 4 | 55.000 | .0 | . | . | 1 | 3 |
|  | 5 | 61.000 | .0 | . | . | 1 | 2 |
|  | 6 | 66.000 | .0 | . | . | 1 | 1 |
|  | 7 | 71.000 | .0 | . | . | 1 | 0 |
| 1.33 | 1 | 17.000 | 1.0 | .957 | .043 | 1 | 22 |
|  | 2 | 22.000 | .0 | . | . | 1 | 21 |
|  | 3 | 24.000 | .0 | . | . | 1 | 20 |
|  | 4 | 25.000 | .0 | . | . | 1 | 19 |
|  | 5 | 26.000 | .0 | . | . | 1 | 18 |
|  | 6 | 27.000 | .0 | . | . | 1 | 17 |
|  | 7 | 29.000 | .0 | . | . | 1 | 16 |
|  | 8 | 41.000 | .0 | . | . | 1 | 15 |
|  | 9 | 44.000 | .0 | . | . | 1 | 14 |
|  | 10 | 44.000 | .0 | . | . | 1 | 13 |
|  | 11 | 46.000 | .0 | . | . | 1 | 12 |
|  | 12 | 48.000 | 1.0 | .877 | .086 | 2 | 11 |
|  | 13 | 48.000 | .0 | . | . | 2 | 10 |
|  | 14 | 49.000 | .0 | . | . | 2 | 9 |
|  | 15 | 50.000 | .0 | . | . | 2 | 8 |
|  | 16 | 51.000 | .0 | . | . | 2 | 7 |
|  | 17 | 54.000 | .0 | . | . | 2 | 6 |
|  | 18 | 59.000 | .0 | . | . | 2 | 5 |
|  | 19 | 66.000 | .0 | . | . | 2 | 4 |
|  | 20 | 67.000 | .0 | . | . | 2 | 3 |
|  | 21 | 67.000 | .0 | . | . | 2 | 2 |
|  | 22 | 68.000 | .0 | . | . | 2 | 1 |
|  | 23 | 73.000 | .0 | . | . | 2 | 0 |
| 1.38 | 1 | 19.000 | 1.0 | .938 | .061 | 1 | 15 |
|  | 2 | 20.000 | .0 | . | . | 1 | 14 |
|  | 3 | 22.000 | .0 | . | . | 1 | 13 |
|  | 4 | 24.000 | .0 | . | . | 1 | 12 |
|  | 5 | 24.000 | .0 | . | . | 1 | 11 |
|  | 6 | 26.000 | .0 | . | . | 1 | 10 |
|  | 7 | 28.000 | .0 | . | . | 1 | 9 |
|  | 8 | 41.000 | 1.0 | .833 | .112 | 2 | 8 |
|  | 9 | 48.000 | .0 | . | . | 2 | 7 |
|  | 10 | 49.000 | .0 | . | . | 2 | 6 |
|  | 11 | 51.000 | .0 | . | . | 2 | 5 |
|  | 12 | 53.000 | .0 | . | . | 2 | 4 |
|  | 13 | 61.000 | .0 | . | . | 2 | 3 |
|  | 14 | 66.000 | .0 | . | . | 2 | 2 |
|  | 15 | 68.000 | .0 | . | . | 2 | 1 |
|  | 16 | 70.000 | .0 | . | . | 2 | 0 |
| 1.41 | 1 | 7.000 | 1.0 | .944 | .054 | 1 | 17 |
|  | 2 | 9.000 | .0 | . | . | 1 | 16 |
|  | 3 | 12.000 | .0 | . | . | 1 | 15 |
|  | 4 | 22.000 | .0 | . | . | 1 | 14 |
|  | 5 | 24.000 | .0 | . | . | 1 | 13 |
|  | 6 | 30.000 | .0 | . | . | 1 | 12 |
|  | 7 | 41.000 | .0 | . | . | 1 | 11 |
|  | 8 | 46.000 | .0 | . | . | 1 | 10 |
|  | 9 | 48.000 | .0 | . | . | 1 | 9 |
|  | 10 | 48.000 | .0 | . | . | 1 | 8 |
|  | 11 | 49.000 | .0 | . | . | 1 | 7 |
|  | 12 | 51.000 | .0 | . | . | 1 | 6 |
|  | 13 | 54.000 | .0 | . | . | 1 | 5 |
|  | 14 | 56.000 | .0 | . | . | 1 | 4 |
|  | 15 | 60.000 | 1.0 | .708 | .208 | 2 | 3 |
|  | 16 | 60.000 | .0 | . | . | 2 | 2 |
|  | 17 | 69.000 | .0 | . | . | 2 | 1 |
|  | 18 | 77.000 | .0 | . | . | 2 | 0 |
| 1.43 | 1 | 24.000 | 1.0 | .929 | .069 | 1 | 13 |
|  | 2 | 25.000 | .0 | . | . | 1 | 12 |
|  | 3 | 27.000 | .0 | . | . | 1 | 11 |
|  | 4 | 29.000 | .0 | . | . | 1 | 10 |
|  | 5 | 34.000 | .0 | . | . | 1 | 9 |
|  | 6 | 49.000 | .0 | . | . | 1 | 8 |
|  | 7 | 53.000 | .0 | . | . | 1 | 7 |
|  | 8 | 54.000 | .0 | . | . | 1 | 6 |
|  | 9 | 60.000 | .0 | . | . | 1 | 5 |
|  | 10 | 60.000 | .0 | . | . | 1 | 4 |
|  | 11 | 63.000 | .0 | . | . | 1 | 3 |
|  | 12 | 65.000 | .0 | . | . | 1 | 2 |
|  | 13 | 71.000 | .0 | . | . | 1 | 1 |
|  | 14 | 76.000 | .0 | . | . | 1 | 0 |
| 1.46 | 1 | 45.000 | 1.0 | .909 | .087 | 1 | 10 |
|  | 2 | 49.000 | .0 | . | . | 1 | 9 |
|  | 3 | 50.000 | .0 | . | . | 1 | 8 |
|  | 4 | 51.000 | .0 | . | . | 1 | 7 |
|  | 5 | 55.000 | .0 | . | . | 1 | 6 |
|  | 6 | 55.000 | .0 | . | . | 1 | 5 |
|  | 7 | 66.000 | .0 | . | . | 1 | 4 |
|  | 8 | 67.000 | .0 | . | . | 1 | 3 |
|  | 9 | 69.000 | .0 | . | . | 1 | 2 |
|  | 10 | 70.000 | .0 | . | . | 1 | 1 |
|  | 11 | 73.000 | .0 | . | . | 1 | 0 |
| 1.47 | 1 | 13.000 | 1.0 | .947 | .051 | 1 | 18 |
|  | 2 | 17.000 | 1.0 | .895 | .070 | 2 | 17 |
|  | 3 | 22.000 | .0 | . | . | 2 | 16 |
|  | 4 | 27.000 | .0 | . | . | 2 | 15 |
|  | 5 | 30.000 | .0 | . | . | 2 | 14 |
|  | 6 | 36.000 | .0 | . | . | 2 | 13 |
|  | 7 | 40.000 | .0 | . | . | 2 | 12 |
|  | 8 | 42.000 | .0 | . | . | 2 | 11 |
|  | 9 | 44.000 | .0 | . | . | 2 | 10 |
|  | 10 | 45.000 | .0 | . | . | 2 | 9 |
|  | 11 | 46.000 | .0 | . | . | 2 | 8 |
|  | 12 | 48.000 | .0 | . | . | 2 | 7 |
|  | 13 | 53.000 | .0 | . | . | 2 | 6 |
|  | 14 | 53.000 | .0 | . | . | 2 | 5 |
|  | 15 | 67.000 | .0 | . | . | 2 | 4 |
|  | 16 | 68.000 | .0 | . | . | 2 | 3 |
|  | 17 | 68.000 | .0 | . | . | 2 | 2 |
|  | 18 | 72.000 | .0 | . | . | 2 | 1 |
|  | 19 | 76.000 | .0 | . | . | 2 | 0 |
| 1.48 | 1 | 34.000 | 1.0 | .938 | .061 | 1 | 15 |
|  | 2 | 38.000 | .0 | . | . | 1 | 14 |
|  | 3 | 50.000 | .0 | . | . | 1 | 13 |
|  | 4 | 50.000 | .0 | . | . | 1 | 12 |
|  | 5 | 50.000 | .0 | . | . | 1 | 11 |
|  | 6 | 51.000 | .0 | . | . | 1 | 10 |
|  | 7 | 54.000 | .0 | . | . | 1 | 9 |
|  | 8 | 56.000 | .0 | . | . | 1 | 8 |
|  | 9 | 60.000 | .0 | . | . | 1 | 7 |
|  | 10 | 67.000 | .0 | . | . | 1 | 6 |
|  | 11 | 68.000 | .0 | . | . | 1 | 5 |
|  | 12 | 70.000 | .0 | . | . | 1 | 4 |
|  | 13 | 72.000 | .0 | . | . | 1 | 3 |
|  | 14 | 74.000 | .0 | . | . | 1 | 2 |
|  | 15 | 74.000 | .0 | . | . | 1 | 1 |
|  | 16 | 76.000 | .0 | . | . | 1 | 0 |
| 1.49 | 1 | 10.000 | 1.0 | .875 | .117 | 1 | 7 |
|  | 2 | 13.000 | .0 | . | . | 1 | 6 |
|  | 3 | 19.000 | .0 | . | . | 1 | 5 |
|  | 4 | 26.000 | .0 | . | . | 1 | 4 |
|  | 5 | 28.000 | .0 | . | . | 1 | 3 |
|  | 6 | 29.000 | .0 | . | . | 1 | 2 |
|  | 7 | 57.000 | .0 | . | . | 1 | 1 |
|  | 8 | 70.000 | .0 | . | . | 1 | 0 |
| 1.50 | 1 | 36.000 | 1.0 | . | . | 1 | 25 |
|  | 2 | 36.000 | 1.0 | .923 | .052 | 2 | 24 |
|  | 3 | 41.000 | .0 | . | . | 2 | 23 |
|  | 4 | 41.000 | .0 | . | . | 2 | 22 |
|  | 5 | 48.000 | .0 | . | . | 2 | 21 |
|  | 6 | 49.000 | .0 | . | . | 2 | 20 |
|  | 7 | 49.000 | .0 | . | . | 2 | 19 |
|  | 8 | 50.000 | .0 | . | . | 2 | 18 |
|  | 9 | 51.000 | .0 | . | . | 2 | 17 |
|  | 10 | 52.000 | .0 | . | . | 2 | 16 |
|  | 11 | 53.000 | .0 | . | . | 2 | 15 |
|  | 12 | 54.000 | .0 | . | . | 2 | 14 |
|  | 13 | 58.000 | .0 | . | . | 2 | 13 |
|  | 14 | 59.000 | .0 | . | . | 2 | 12 |
|  | 15 | 59.000 | .0 | . | . | 2 | 11 |
|  | 16 | 63.000 | .0 | . | . | 2 | 10 |
|  | 17 | 63.000 | .0 | . | . | 2 | 9 |
|  | 18 | 66.000 | .0 | . | . | 2 | 8 |
|  | 19 | 67.000 | .0 | . | . | 2 | 7 |
|  | 20 | 69.000 | .0 | . | . | 2 | 6 |
|  | 21 | 70.000 | .0 | . | . | 2 | 5 |
|  | 22 | 71.000 | .0 | . | . | 2 | 4 |
|  | 23 | 71.000 | .0 | . | . | 2 | 3 |
|  | 24 | 74.000 | .0 | . | . | 2 | 2 |
|  | 25 | 75.000 | .0 | . | . | 2 | 1 |
|  | 26 | 76.000 | .0 | . | . | 2 | 0 |
| 1.53 | 1 | 4.000 | 1.0 | .941 | .057 | 1 | 16 |
|  | 2 | 6.000 | .0 | . | . | 1 | 15 |
|  | 3 | 14.000 | .0 | . | . | 1 | 14 |
|  | 4 | 18.000 | .0 | . | . | 1 | 13 |
|  | 5 | 20.000 | .0 | . | . | 1 | 12 |
|  | 6 | 24.000 | .0 | . | . | 1 | 11 |
|  | 7 | 26.000 | .0 | . | . | 1 | 10 |
|  | 8 | 29.000 | .0 | . | . | 1 | 9 |
|  | 9 | 30.000 | .0 | . | . | 1 | 8 |
|  | 10 | 46.000 | .0 | . | . | 1 | 7 |
|  | 11 | 47.000 | .0 | . | . | 1 | 6 |
|  | 12 | 47.000 | .0 | . | . | 1 | 5 |
|  | 13 | 48.000 | .0 | . | . | 1 | 4 |
|  | 14 | 51.000 | .0 | . | . | 1 | 3 |
|  | 15 | 52.000 | .0 | . | . | 1 | 2 |
|  | 16 | 68.000 | .0 | . | . | 1 | 1 |
|  | 17 | 73.000 | .0 | . | . | 1 | 0 |
| 1.54 | 1 | 24.000 | 1.0 | .929 | .069 | 1 | 13 |
|  | 2 | 25.000 | .0 | . | . | 1 | 12 |
|  | 3 | 27.000 | .0 | . | . | 1 | 11 |
|  | 4 | 38.000 | .0 | . | . | 1 | 10 |
|  | 5 | 41.000 | .0 | . | . | 1 | 9 |
|  | 6 | 48.000 | 1.0 | .825 | .115 | 2 | 8 |
|  | 7 | 50.000 | .0 | . | . | 2 | 7 |
|  | 8 | 52.000 | .0 | . | . | 2 | 6 |
|  | 9 | 56.000 | .0 | . | . | 2 | 5 |
|  | 10 | 56.000 | .0 | . | . | 2 | 4 |
|  | 11 | 59.000 | .0 | . | . | 2 | 3 |
|  | 12 | 65.000 | .0 | . | . | 2 | 2 |
|  | 13 | 67.000 | .0 | . | . | 2 | 1 |
|  | 14 | 68.000 | .0 | . | . | 2 | 0 |
| 1.55 | 1 | 45.000 | 1.0 | .857 | .132 | 1 | 6 |
|  | 2 | 48.000 | .0 | . | . | 1 | 5 |
|  | 3 | 48.000 | .0 | . | . | 1 | 4 |
|  | 4 | 51.000 | .0 | . | . | 1 | 3 |
|  | 5 | 52.000 | .0 | . | . | 1 | 2 |
|  | 6 | 52.000 | .0 | . | . | 1 | 1 |
|  | 7 | 70.000 | .0 | . | . | 1 | 0 |
| 1.57 | 1 | 9.000 | 1.0 | .950 | .049 | 1 | 19 |
|  | 2 | 12.000 | .0 | . | . | 1 | 18 |
|  | 3 | 12.000 | .0 | . | . | 1 | 17 |
|  | 4 | 13.000 | .0 | . | . | 1 | 16 |
|  | 5 | 22.000 | .0 | . | . | 1 | 15 |
|  | 6 | 22.000 | .0 | . | . | 1 | 14 |
|  | 7 | 23.000 | .0 | . | . | 1 | 13 |
|  | 8 | 25.000 | .0 | . | . | 1 | 12 |
|  | 9 | 40.000 | 1.0 | .871 | .088 | 2 | 11 |
|  | 10 | 44.000 | .0 | . | . | 2 | 10 |
|  | 11 | 48.000 | 1.0 | .784 | .114 | 3 | 9 |
|  | 12 | 50.000 | .0 | . | . | 3 | 8 |
|  | 13 | 52.000 | .0 | . | . | 3 | 7 |
|  | 14 | 55.000 | .0 | . | . | 3 | 6 |
|  | 15 | 66.000 | .0 | . | . | 3 | 5 |
|  | 16 | 67.000 | .0 | . | . | 3 | 4 |
|  | 17 | 68.000 | .0 | . | . | 3 | 3 |
|  | 18 | 70.000 | .0 | . | . | 3 | 2 |
|  | 19 | 77.000 | .0 | . | . | 3 | 1 |
|  | 20 | 78.000 | .0 | . | . | 3 | 0 |
| 1.59 | 1 | 36.000 | 1.0 | .909 | .087 | 1 | 10 |
|  | 2 | 38.000 | .0 | . | . | 1 | 9 |
|  | 3 | 45.000 | 1.0 | .808 | .122 | 2 | 8 |
|  | 4 | 45.000 | .0 | . | . | 2 | 7 |
|  | 5 | 48.000 | .0 | . | . | 2 | 6 |
|  | 6 | 52.000 | .0 | . | . | 2 | 5 |
|  | 7 | 60.000 | .0 | . | . | 2 | 4 |
|  | 8 | 65.000 | .0 | . | . | 2 | 3 |
|  | 9 | 72.000 | .0 | . | . | 2 | 2 |
|  | 10 | 74.000 | .0 | . | . | 2 | 1 |
|  | 11 | 75.000 | .0 | . | . | 2 | 0 |
| 1.62 | 1 | 48.000 | 1.0 | .800 | .179 | 1 | 4 |
|  | 2 | 54.000 | .0 | . | . | 1 | 3 |
|  | 3 | 59.000 | .0 | . | . | 1 | 2 |
|  | 4 | 71.000 | .0 | . | . | 1 | 1 |
|  | 5 | 75.000 | .0 | . | . | 1 | 0 |
| 1.63 | 1 | 36.000 | 1.0 | .909 | .087 | 1 | 10 |
|  | 2 | 45.000 | .0 | . | . | 1 | 9 |
|  | 3 | 49.000 | .0 | . | . | 1 | 8 |
|  | 4 | 51.000 | .0 | . | . | 1 | 7 |
|  | 5 | 52.000 | .0 | . | . | 1 | 6 |
|  | 6 | 54.000 | .0 | . | . | 1 | 5 |
|  | 7 | 57.000 | .0 | . | . | 1 | 4 |
|  | 8 | 59.000 | .0 | . | . | 1 | 3 |
|  | 9 | 60.000 | .0 | . | . | 1 | 2 |
|  | 10 | 68.000 | .0 | . | . | 1 | 1 |
|  | 11 | 69.000 | .0 | . | . | 1 | 0 |
| 1.64 | 1 | 10.000 | 1.0 | .938 | .061 | 1 | 15 |
|  | 2 | 12.000 | .0 | . | . | 1 | 14 |
|  | 3 | 20.000 | .0 | . | . | 1 | 13 |
|  | 4 | 22.000 | .0 | . | . | 1 | 12 |
|  | 5 | 42.000 | .0 | . | . | 1 | 11 |
|  | 6 | 43.000 | .0 | . | . | 1 | 10 |
|  | 7 | 45.000 | .0 | . | . | 1 | 9 |
|  | 8 | 47.000 | .0 | . | . | 1 | 8 |
|  | 9 | 47.000 | .0 | . | . | 1 | 7 |
|  | 10 | 54.000 | .0 | . | . | 1 | 6 |
|  | 11 | 57.000 | .0 | . | . | 1 | 5 |
|  | 12 | 57.000 | .0 | . | . | 1 | 4 |
|  | 13 | 61.000 | .0 | . | . | 1 | 3 |
|  | 14 | 71.000 | .0 | . | . | 1 | 2 |
|  | 15 | 71.000 | .0 | . | . | 1 | 1 |
|  | 16 | 76.000 | .0 | . | . | 1 | 0 |
| 1.67 | 1 | 45.000 | 1.0 | .909 | .087 | 1 | 10 |
|  | 2 | 45.000 | .0 | . | . | 1 | 9 |
|  | 3 | 45.000 | .0 | . | . | 1 | 8 |
|  | 4 | 46.000 | .0 | . | . | 1 | 7 |
|  | 5 | 67.000 | .0 | . | . | 1 | 6 |
|  | 6 | 68.000 | .0 | . | . | 1 | 5 |
|  | 7 | 69.000 | .0 | . | . | 1 | 4 |
|  | 8 | 71.000 | .0 | . | . | 1 | 3 |
|  | 9 | 73.000 | .0 | . | . | 1 | 2 |
|  | 10 | 73.000 | .0 | . | . | 1 | 1 |
|  | 11 | 74.000 | .0 | . | . | 1 | 0 |
| 1.70 | 1 | 9.000 | 1.0 | .955 | .044 | 1 | 21 |
|  | 2 | 13.000 | .0 | . | . | 1 | 20 |
|  | 3 | 19.000 | .0 | . | . | 1 | 19 |
|  | 4 | 20.000 | .0 | . | . | 1 | 18 |
|  | 5 | 20.000 | .0 | . | . | 1 | 17 |
|  | 6 | 22.000 | .0 | . | . | 1 | 16 |
|  | 7 | 24.000 | .0 | . | . | 1 | 15 |
|  | 8 | 25.000 | .0 | . | . | 1 | 14 |
|  | 9 | 28.000 | .0 | . | . | 1 | 13 |
|  | 10 | 36.000 | .0 | . | . | 1 | 12 |
|  | 11 | 40.000 | .0 | . | . | 1 | 11 |
|  | 12 | 46.000 | .0 | . | . | 1 | 10 |
|  | 13 | 49.000 | .0 | . | . | 1 | 9 |
|  | 14 | 50.000 | .0 | . | . | 1 | 8 |
|  | 15 | 53.000 | .0 | . | . | 1 | 7 |
|  | 16 | 55.000 | .0 | . | . | 1 | 6 |
|  | 17 | 56.000 | .0 | . | . | 1 | 5 |
|  | 18 | 68.000 | .0 | . | . | 1 | 4 |
|  | 19 | 71.000 | .0 | . | . | 1 | 3 |
|  | 20 | 71.000 | .0 | . | . | 1 | 2 |
|  | 21 | 72.000 | .0 | . | . | 1 | 1 |
|  | 22 | 72.000 | .0 | . | . | 1 | 0 |
| 1.73 | 1 | 4.000 | 1.0 | .952 | .046 | 1 | 20 |
|  | 2 | 15.000 | .0 | . | . | 1 | 19 |
|  | 3 | 18.000 | .0 | . | . | 1 | 18 |
|  | 4 | 20.000 | .0 | . | . | 1 | 17 |
|  | 5 | 21.000 | .0 | . | . | 1 | 16 |
|  | 6 | 21.000 | .0 | . | . | 1 | 15 |
|  | 7 | 23.000 | .0 | . | . | 1 | 14 |
|  | 8 | 26.000 | .0 | . | . | 1 | 13 |
|  | 9 | 27.000 | .0 | . | . | 1 | 12 |
|  | 10 | 29.000 | .0 | . | . | 1 | 11 |
|  | 11 | 38.000 | .0 | . | . | 1 | 10 |
|  | 12 | 40.000 | .0 | . | . | 1 | 9 |
|  | 13 | 41.000 | .0 | . | . | 1 | 8 |
|  | 14 | 42.000 | .0 | . | . | 1 | 7 |
|  | 15 | 43.000 | .0 | . | . | 1 | 6 |
|  | 16 | 47.000 | .0 | . | . | 1 | 5 |
|  | 17 | 48.000 | .0 | . | . | 1 | 4 |
|  | 18 | 56.000 | .0 | . | . | 1 | 3 |
|  | 19 | 60.000 | .0 | . | . | 1 | 2 |
|  | 20 | 74.000 | .0 | . | . | 1 | 1 |
|  | 21 | 77.000 | .0 | . | . | 1 | 0 |
| 1.75 | 1 | 12.000 | 1.0 | .950 | .049 | 1 | 19 |
|  | 2 | 12.000 | .0 | . | . | 1 | 18 |
|  | 3 | 12.000 | .0 | . | . | 1 | 17 |
|  | 4 | 12.000 | .0 | . | . | 1 | 16 |
|  | 5 | 20.000 | .0 | . | . | 1 | 15 |
|  | 6 | 23.000 | .0 | . | . | 1 | 14 |
|  | 7 | 24.000 | .0 | . | . | 1 | 13 |
|  | 8 | 27.000 | .0 | . | . | 1 | 12 |
|  | 9 | 34.000 | .0 | . | . | 1 | 11 |
|  | 10 | 42.000 | .0 | . | . | 1 | 10 |
|  | 11 | 43.000 | .0 | . | . | 1 | 9 |
|  | 12 | 43.000 | .0 | . | . | 1 | 8 |
|  | 13 | 46.000 | .0 | . | . | 1 | 7 |
|  | 14 | 50.000 | .0 | . | . | 1 | 6 |
|  | 15 | 54.000 | .0 | . | . | 1 | 5 |
|  | 16 | 56.000 | .0 | . | . | 1 | 4 |
|  | 17 | 59.000 | .0 | . | . | 1 | 3 |
|  | 18 | 63.000 | 1.0 | .633 | .261 | 2 | 2 |
|  | 19 | 69.000 | .0 | . | . | 2 | 1 |
|  | 20 | 74.000 | .0 | . | . | 2 | 0 |
| 1.76 | 1 | 43.000 | 1.0 | .909 | .087 | 1 | 10 |
|  | 2 | 47.000 | .0 | . | . | 1 | 9 |
|  | 3 | 50.000 | .0 | . | . | 1 | 8 |
|  | 4 | 52.000 | .0 | . | . | 1 | 7 |
|  | 5 | 52.000 | .0 | . | . | 1 | 6 |
|  | 6 | 67.000 | 1.0 | .758 | .156 | 2 | 5 |
|  | 7 | 68.000 | .0 | . | . | 2 | 4 |
|  | 8 | 68.000 | .0 | . | . | 2 | 3 |
|  | 9 | 69.000 | 1.0 | .505 | .231 | 3 | 2 |
|  | 10 | 77.000 | .0 | . | . | 3 | 1 |
|  | 11 | 77.000 | .0 | . | . | 3 | 0 |
| 1.79 | 1 | 16.000 | 1.0 | .933 | .064 | 1 | 14 |
|  | 2 | 22.000 | .0 | . | . | 1 | 13 |
|  | 3 | 22.000 | .0 | . | . | 1 | 12 |
|  | 4 | 24.000 | 1.0 | .856 | .095 | 2 | 11 |
|  | 5 | 24.000 | .0 | . | . | 2 | 10 |
|  | 6 | 24.000 | .0 | . | . | 2 | 9 |
|  | 7 | 26.000 | .0 | . | . | 2 | 8 |
|  | 8 | 28.000 | .0 | . | . | 2 | 7 |
|  | 9 | 47.000 | .0 | . | . | 2 | 6 |
|  | 10 | 47.000 | .0 | . | . | 2 | 5 |
|  | 11 | 51.000 | .0 | . | . | 2 | 4 |
|  | 12 | 52.000 | .0 | . | . | 2 | 3 |
|  | 13 | 56.000 | .0 | . | . | 2 | 2 |
|  | 14 | 70.000 | .0 | . | . | 2 | 1 |
|  | 15 | 72.000 | .0 | . | . | 2 | 0 |
| 1.84 | 1 | 48.000 | 1.0 | .667 | .272 | 1 | 2 |
|  | 2 | 57.000 | .0 | . | . | 1 | 1 |
|  | 3 | 64.000 | .0 | . | . | 1 | 0 |
| 1.88 | 1 | 24.000 | 1.0 | .944 | .054 | 1 | 17 |
|  | 2 | 24.000 | .0 | . | . | 1 | 16 |
|  | 3 | 26.000 | .0 | . | . | 1 | 15 |
|  | 4 | 26.000 | .0 | . | . | 1 | 14 |
|  | 5 | 29.000 | .0 | . | . | 1 | 13 |
|  | 6 | 48.000 | .0 | . | . | 1 | 12 |
|  | 7 | 48.000 | .0 | . | . | 1 | 11 |
|  | 8 | 50.000 | .0 | . | . | 1 | 10 |
|  | 9 | 51.000 | .0 | . | . | 1 | 9 |
|  | 10 | 52.000 | .0 | . | . | 1 | 8 |
|  | 11 | 56.000 | .0 | . | . | 1 | 7 |
|  | 12 | 57.000 | .0 | . | . | 1 | 6 |
|  | 13 | 61.000 | .0 | . | . | 1 | 5 |
|  | 14 | 64.000 | .0 | . | . | 1 | 4 |
|  | 15 | 66.000 | .0 | . | . | 1 | 3 |
|  | 16 | 68.000 | .0 | . | . | 1 | 2 |
|  | 17 | 69.000 | .0 | . | . | 1 | 1 |
|  | 18 | 70.000 | .0 | . | . | 1 | 0 |
| 1.90 | 1 | 15.000 | 1.0 | .923 | .074 | 1 | 12 |
|  | 2 | 18.000 | .0 | . | . | 1 | 11 |
|  | 3 | 22.000 | .0 | . | . | 1 | 10 |
|  | 4 | 24.000 | .0 | . | . | 1 | 9 |
|  | 5 | 29.000 | .0 | . | . | 1 | 8 |
|  | 6 | 41.000 | .0 | . | . | 1 | 7 |
|  | 7 | 41.000 | .0 | . | . | 1 | 6 |
|  | 8 | 45.000 | .0 | . | . | 1 | 5 |
|  | 9 | 48.000 | .0 | . | . | 1 | 4 |
|  | 10 | 49.000 | .0 | . | . | 1 | 3 |
|  | 11 | 50.000 | .0 | . | . | 1 | 2 |
|  | 12 | 58.000 | .0 | . | . | 1 | 1 |
|  | 13 | 59.000 | .0 | . | . | 1 | 0 |
| 1.92 | 1 | 26.000 | 1.0 | .750 | .217 | 1 | 3 |
|  | 2 | 53.000 | .0 | . | . | 1 | 2 |
|  | 3 | 55.000 | .0 | . | . | 1 | 1 |
|  | 4 | 74.000 | .0 | . | . | 1 | 0 |
| 1.94 | 1 | 3.000 | 1.0 | .875 | .117 | 1 | 7 |
|  | 2 | 18.000 | .0 | . | . | 1 | 6 |
|  | 3 | 20.000 | .0 | . | . | 1 | 5 |
|  | 4 | 22.000 | .0 | . | . | 1 | 4 |
|  | 5 | 24.000 | .0 | . | . | 1 | 3 |
|  | 6 | 28.000 | .0 | . | . | 1 | 2 |
|  | 7 | 44.000 | .0 | . | . | 1 | 1 |
|  | 8 | 58.000 | .0 | . | . | 1 | 0 |
| 2.00 | 1 | 24.000 | 1.0 | .958 | .041 | 1 | 23 |
|  | 2 | 26.000 | .0 | . | . | 1 | 22 |
|  | 3 | 36.000 | .0 | . | . | 1 | 21 |
|  | 4 | 38.000 | .0 | . | . | 1 | 20 |
|  | 5 | 40.000 | .0 | . | . | 1 | 19 |
|  | 6 | 44.000 | .0 | . | . | 1 | 18 |
|  | 7 | 45.000 | .0 | . | . | 1 | 17 |
|  | 8 | 45.000 | .0 | . | . | 1 | 16 |
|  | 9 | 47.000 | .0 | . | . | 1 | 15 |
|  | 10 | 47.000 | .0 | . | . | 1 | 14 |
|  | 11 | 47.000 | .0 | . | . | 1 | 13 |
|  | 12 | 48.000 | .0 | . | . | 1 | 12 |
|  | 13 | 48.000 | .0 | . | . | 1 | 11 |
|  | 14 | 49.000 | .0 | . | . | 1 | 10 |
|  | 15 | 49.000 | .0 | . | . | 1 | 9 |
|  | 16 | 51.000 | .0 | . | . | 1 | 8 |
|  | 17 | 52.000 | .0 | . | . | 1 | 7 |
|  | 18 | 69.000 | .0 | . | . | 1 | 6 |
|  | 19 | 71.000 | .0 | . | . | 1 | 5 |
|  | 20 | 72.000 | .0 | . | . | 1 | 4 |
|  | 21 | 72.000 | .0 | . | . | 1 | 3 |
|  | 22 | 72.000 | .0 | . | . | 1 | 2 |
|  | 23 | 74.000 | .0 | . | . | 1 | 1 |
|  | 24 | 74.000 | .0 | . | . | 1 | 0 |
| 2.04 | 1 | 36.000 | 1.0 | .857 | .132 | 1 | 6 |
|  | 2 | 48.000 | .0 | . | . | 1 | 5 |
|  | 3 | 49.000 | .0 | . | . | 1 | 4 |
|  | 4 | 49.000 | .0 | . | . | 1 | 3 |
|  | 5 | 50.000 | .0 | . | . | 1 | 2 |
|  | 6 | 58.000 | .0 | . | . | 1 | 1 |
|  | 7 | 75.000 | .0 | . | . | 1 | 0 |
| 2.08 | 1 | 1.000 | 1.0 | .889 | .105 | 1 | 8 |
|  | 2 | 1.000 | .0 | . | . | 1 | 7 |
|  | 3 | 3.000 | .0 | . | . | 1 | 6 |
|  | 4 | 20.000 | .0 | . | . | 1 | 5 |
|  | 5 | 26.000 | .0 | . | . | 1 | 4 |
|  | 6 | 28.000 | .0 | . | . | 1 | 3 |
|  | 7 | 28.000 | .0 | . | . | 1 | 2 |
|  | 8 | 35.000 | .0 | . | . | 1 | 1 |
|  | 9 | 55.000 | .0 | . | . | 1 | 0 |
| 2.10 | 1 | 3.000 | 1.0 | .917 | .080 | 1 | 11 |
|  | 2 | 12.000 | .0 | . | . | 1 | 10 |
|  | 3 | 12.000 | .0 | . | . | 1 | 9 |
|  | 4 | 19.000 | .0 | . | . | 1 | 8 |
|  | 5 | 20.000 | .0 | . | . | 1 | 7 |
|  | 6 | 24.000 | .0 | . | . | 1 | 6 |
|  | 7 | 27.000 | .0 | . | . | 1 | 5 |
|  | 8 | 29.000 | .0 | . | . | 1 | 4 |
|  | 9 | 46.000 | .0 | . | . | 1 | 3 |
|  | 10 | 47.000 | .0 | . | . | 1 | 2 |
|  | 11 | 55.000 | .0 | . | . | 1 | 1 |
|  | 12 | 73.000 | .0 | . | . | 1 | 0 |
| 2.12 | 1 | 36.000 | 1.0 | .875 | .117 | 1 | 7 |
|  | 2 | 44.000 | .0 | . | . | 1 | 6 |
|  | 3 | 46.000 | .0 | . | . | 1 | 5 |
|  | 4 | 51.000 | .0 | . | . | 1 | 4 |
|  | 5 | 55.000 | .0 | . | . | 1 | 3 |
|  | 6 | 56.000 | .0 | . | . | 1 | 2 |
|  | 7 | 71.000 | .0 | . | . | 1 | 1 |
|  | 8 | 74.000 | .0 | . | . | 1 | 0 |
| 2.17 | 1 | 17.000 | 1.0 | .833 | .152 | 1 | 5 |
|  | 2 | 27.000 | .0 | . | . | 1 | 4 |
|  | 3 | 29.000 | .0 | . | . | 1 | 3 |
|  | 4 | 29.000 | .0 | . | . | 1 | 2 |
|  | 5 | 60.000 | .0 | . | . | 1 | 1 |
|  | 6 | 74.000 | .0 | . | . | 1 | 0 |
| 2.21 | 1 | 3.000 | 1.0 | .900 | .095 | 1 | 9 |
|  | 2 | 9.000 | .0 | . | . | 1 | 8 |
|  | 3 | 13.000 | .0 | . | . | 1 | 7 |
|  | 4 | 16.000 | .0 | . | . | 1 | 6 |
|  | 5 | 19.000 | .0 | . | . | 1 | 5 |
|  | 6 | 28.000 | .0 | . | . | 1 | 4 |
|  | 7 | 46.000 | .0 | . | . | 1 | 3 |
|  | 8 | 48.000 | .0 | . | . | 1 | 2 |
|  | 9 | 49.000 | .0 | . | . | 1 | 1 |
|  | 10 | 75.000 | .0 | . | . | 1 | 0 |
| 2.25 | 1 | 13.000 | 1.0 | .857 | .132 | 1 | 6 |
|  | 2 | 30.000 | 1.0 | .714 | .171 | 2 | 5 |
|  | 3 | 32.000 | .0 | . | . | 2 | 4 |
|  | 4 | 46.000 | .0 | . | . | 2 | 3 |
|  | 5 | 48.000 | .0 | . | . | 2 | 2 |
|  | 6 | 51.000 | .0 | . | . | 2 | 1 |
|  | 7 | 52.000 | .0 | . | . | 2 | 0 |
| 2.32 | 1 | 64.000 | 1.0 | .500 | .354 | 1 | 1 |
|  | 2 | 66.000 | .0 | . | . | 1 | 0 |
| 2.40 | 1 | 48.000 | 1.0 | .500 | .354 | 1 | 1 |
|  | 2 | 63.000 | .0 | . | . | 1 | 0 |
| 2.48 | 1 | 36.000 | 1.0 | .750 | .217 | 1 | 3 |
|  | 2 | 36.000 | .0 | . | . | 1 | 2 |
|  | 3 | 49.000 | .0 | . | . | 1 | 1 |
|  | 4 | 69.000 | .0 | . | . | 1 | 0 |
| 2.49 | 1 | 18.000 | 1.0 | .750 | .217 | 1 | 3 |
|  | 2 | 44.000 | .0 | . | . | 1 | 2 |
|  | 3 | 45.000 | .0 | . | . | 1 | 1 |
|  | 4 | 46.000 | .0 | . | . | 1 | 0 |
| 2.54 | 1 | 18.000 | 1.0 | .875 | .117 | 1 | 7 |
|  | 2 | 20.000 | .0 | . | . | 1 | 6 |
|  | 3 | 22.000 | .0 | . | . | 1 | 5 |
|  | 4 | 48.000 | .0 | . | . | 1 | 4 |
|  | 5 | 56.000 | 1.0 | .656 | .209 | 2 | 3 |
|  | 6 | 62.000 | .0 | . | . | 2 | 2 |
|  | 7 | 65.000 | .0 | . | . | 2 | 1 |
|  | 8 | 67.000 | 1.0 | .000 | .000 | 3 | 0 |
| 2.55 | 1 | 12.000 | 1.0 | .750 | .217 | 1 | 3 |
|  | 2 | 48.000 | .0 | . | . | 1 | 2 |
|  | 3 | 67.000 | .0 | . | . | 1 | 1 |
|  | 4 | 72.000 | .0 | . | . | 1 | 0 |
| 2.58 | 1 | 18.000 | 1.0 | .875 | .117 | 1 | 7 |
|  | 2 | 20.000 | .0 | . | . | 1 | 6 |
|  | 3 | 21.000 | .0 | . | . | 1 | 5 |
|  | 4 | 28.000 | .0 | . | . | 1 | 4 |
|  | 5 | 41.000 | .0 | . | . | 1 | 3 |
|  | 6 | 46.000 | .0 | . | . | 1 | 2 |
|  | 7 | 51.000 | .0 | . | . | 1 | 1 |
|  | 8 | 76.000 | .0 | . | . | 1 | 0 |
| 2.59 | 1 | 48.000 | 1.0 | .667 | .272 | 1 | 2 |
|  | 2 | 48.000 | .0 | . | . | 1 | 1 |
|  | 3 | 51.000 | .0 | . | . | 1 | 0 |
| 2.63 | 1 | 12.000 | 1.0 | .900 | .095 | 1 | 9 |
|  | 2 | 22.000 | .0 | . | . | 1 | 8 |
|  | 3 | 23.000 | .0 | . | . | 1 | 7 |
|  | 4 | 29.000 | .0 | . | . | 1 | 6 |
|  | 5 | 30.000 | .0 | . | . | 1 | 5 |
|  | 6 | 36.000 | 1.0 | .720 | .178 | 2 | 4 |
|  | 7 | 38.000 | .0 | . | . | 2 | 3 |
|  | 8 | 46.000 | .0 | . | . | 2 | 2 |
|  | 9 | 46.000 | .0 | . | . | 2 | 1 |
|  | 10 | 49.000 | .0 | . | . | 2 | 0 |
| 2.64 | 1 | 51.000 | 1.0 | .750 | .217 | 1 | 3 |
|  | 2 | 58.000 | .0 | . | . | 1 | 2 |
|  | 3 | 74.000 | .0 | . | . | 1 | 1 |
|  | 4 | 75.000 | .0 | . | . | 1 | 0 |
| 2.68 | 1 | 48.000 | 1.0 | .857 | .132 | 1 | 6 |
|  | 2 | 48.000 | .0 | . | . | 1 | 5 |
|  | 3 | 48.000 | .0 | . | . | 1 | 4 |
|  | 4 | 51.000 | .0 | . | . | 1 | 3 |
|  | 5 | 58.000 | .0 | . | . | 1 | 2 |
|  | 6 | 64.000 | .0 | . | . | 1 | 1 |
|  | 7 | 76.000 | .0 | . | . | 1 | 0 |
| 2.71 | 1 | 24.000 | 1.0 | .857 | .132 | 1 | 6 |
|  | 2 | 24.000 | .0 | . | . | 1 | 5 |
|  | 3 | 25.000 | .0 | . | . | 1 | 4 |
|  | 4 | 27.000 | .0 | . | . | 1 | 3 |
|  | 5 | 41.000 | .0 | . | . | 1 | 2 |
|  | 6 | 49.000 | .0 | . | . | 1 | 1 |
|  | 7 | 55.000 | .0 | . | . | 1 | 0 |
| 2.74 | 1 | 12.000 | 1.0 | .857 | .132 | 1 | 6 |
|  | 2 | 12.000 | .0 | . | . | 1 | 5 |
|  | 3 | 18.000 | .0 | . | . | 1 | 4 |
|  | 4 | 21.000 | .0 | . | . | 1 | 3 |
|  | 5 | 55.000 | .0 | . | . | 1 | 2 |
|  | 6 | 63.000 | .0 | . | . | 1 | 1 |
|  | 7 | 66.000 | .0 | . | . | 1 | 0 |
| 2.80 | 1 | 39.000 | 1.0 | .800 | .179 | 1 | 4 |
|  | 2 | 68.000 | .0 | . | . | 1 | 3 |
|  | 3 | 70.000 | .0 | . | . | 1 | 2 |
|  | 4 | 71.000 | .0 | . | . | 1 | 1 |
|  | 5 | 77.000 | .0 | . | . | 1 | 0 |
| 2.82 | 1 | 36.000 | 1.0 | .800 | .179 | 1 | 4 |
|  | 2 | 49.000 | .0 | . | . | 1 | 3 |
|  | 3 | 49.000 | .0 | . | . | 1 | 2 |
|  | 4 | 50.000 | .0 | . | . | 1 | 1 |
|  | 5 | 51.000 | .0 | . | . | 1 | 0 |
| 2.86 | 1 | 48.000 | 1.0 | .667 | .272 | 1 | 2 |
|  | 2 | 69.000 | .0 | . | . | 1 | 1 |
|  | 3 | 71.000 | .0 | . | . | 1 | 0 |
| 2.96 | 1 | 7.000 | 1.0 | .500 | .354 | 1 | 1 |
|  | 2 | 77.000 | .0 | . | . | 1 | 0 |
| 3.05 | 1 | 40.000 | 1.0 | .800 | .179 | 1 | 4 |
|  | 2 | 42.000 | .0 | . | . | 1 | 3 |
|  | 3 | 43.000 | .0 | . | . | 1 | 2 |
|  | 4 | 45.000 | .0 | . | . | 1 | 1 |
|  | 5 | 52.000 | 1.0 | .000 | .000 | 2 | 0 |
| 3.07 | 1 | 12.000 | 1.0 | .800 | .179 | 1 | 4 |
|  | 2 | 47.000 | .0 | . | . | 1 | 3 |
|  | 3 | 57.000 | .0 | . | . | 1 | 2 |
|  | 4 | 68.000 | .0 | . | . | 1 | 1 |
|  | 5 | 76.000 | .0 | . | . | 1 | 0 |
| 3.08 | 1 | 13.000 | 1.0 | .667 | .272 | 1 | 2 |
|  | 2 | 72.000 | .0 | . | . | 1 | 1 |
|  | 3 | 76.000 | .0 | . | . | 1 | 0 |
| 3.10 | 1 | 24.000 | 1.0 | .750 | .217 | 1 | 3 |
|  | 2 | 67.000 | .0 | . | . | 1 | 2 |
|  | 3 | 70.000 | .0 | . | . | 1 | 1 |
|  | 4 | 76.000 | .0 | . | . | 1 | 0 |
| 3.13 | 1 | 42.000 | 1.0 | .667 | .272 | 1 | 2 |
|  | 2 | 48.000 | 1.0 | .333 | .272 | 2 | 1 |
|  | 3 | 75.000 | .0 | . | . | 2 | 0 |
| 3.21 | 1 | 7.000 | 1.0 | .500 | .354 | 1 | 1 |
|  | 2 | 77.000 | .0 | . | . | 1 | 0 |
| 3.33 | 1 | 17.000 | 1.0 | .800 | .179 | 1 | 4 |
|  | 2 | 23.000 | .0 | . | . | 1 | 3 |
|  | 3 | 25.000 | .0 | . | . | 1 | 2 |
|  | 4 | 26.000 | .0 | . | . | 1 | 1 |
|  | 5 | 49.000 | .0 | . | . | 1 | 0 |
| 3.35 | 1 | 10.000 | 1.0 | .750 | .217 | 1 | 3 |
|  | 2 | 28.000 | .0 | . | . | 1 | 2 |
|  | 3 | 60.000 | .0 | . | . | 1 | 1 |
|  | 4 | 74.000 | .0 | . | . | 1 | 0 |
| 3.56 | 1 | 12.000 | 1.0 | .750 | .217 | 1 | 3 |
|  | 2 | 34.000 | .0 | . | . | 1 | 2 |
|  | 3 | 45.000 | .0 | . | . | 1 | 1 |
|  | 4 | 72.000 | .0 | . | . | 1 | 0 |
| 3.63 | 1 | 11.000 | 1.0 | .500 | .354 | 1 | 1 |
|  | 2 | 67.000 | .0 | . | . | 1 | 0 |
| 3.68 | 1 | 44.000 | 1.0 | .000 | .000 | 1 | 0 |
| 3.86 | 1 | 48.000 | 1.0 | .000 | .000 | 1 | 0 |
| 3.90 | 1 | 36.000 | 1.0 | .500 | .354 | 1 | 1 |
|  | 2 | 51.000 | .0 | . | . | 1 | 0 |
| 3.94 | 1 | 6.000 | 1.0 | .000 | .000 | 1 | 0 |
| 3.96 | 1 | 48.000 | 1.0 | .000 | .000 | 1 | 0 |
| 4.14 | 1 | 54.000 | 1.0 | .000 | .000 | 1 | 0 |
| 4.18 | 1 | 3.000 | 1.0 | .750 | .217 | 1 | 3 |
|  | 2 | 34.000 | .0 | . | . | 1 | 2 |
|  | 3 | 34.000 | .0 | . | . | 1 | 1 |
|  | 4 | 76.000 | .0 | . | . | 1 | 0 |
| 4.21 | 1 | 25.000 | 1.0 | .000 | .000 | 1 | 0 |
| 5.00 | 1 | 1.000 | 1.0 | .750 | .217 | 1 | 3 |
|  | 2 | 13.000 | .0 | . | . | 1 | 2 |
|  | 3 | 41.000 | .0 | . | . | 1 | 1 |
|  | 4 | 48.000 | .0 | . | . | 1 | 0 |
| 5.10 | 1 | 24.000 | 1.0 | .000 | .000 | 1 | 0 |
| 5.25 | 1 | 29.000 | 1.0 | .500 | .354 | 1 | 1 |
|  | 2 | 60.000 | 1.0 | .000 | .000 | 2 | 0 |
| 5.28 | 1 | 48.000 | 1.0 | .000 | .000 | 1 | 0 |
| 6.20 | 1 | 1.000 | 1.0 | .500 | .354 | 1 | 1 |
|  | 2 | 1.000 | .0 | . | . | 1 | 0 |
| 8.75 | 1 | 52.000 | 1.0 | .500 | .354 | 1 | 1 |
|  | 2 | 55.000 | .0 | . | . | 1 | 0 |
| 10.24 | 1 | 1.000 | 1.0 | .000 | .000 | 1 | 0 |
| 56.00 | 1 | 24.000 | 1.0 | .000 | .000 | 1 | 0 |
